# Adhesives with Debonding‐On‐Demand Capability: Leveraging Responsive Microcapsules for Mechanically‐Induced Debonding

**DOI:** 10.1002/adma.202414308

**Published:** 2025-03-03

**Authors:** Claas‐Hendrik Stamp, Jana Stumpp, Céline Calvino

**Affiliations:** ^1^ Cluster of Excellence livMatS Albert Ludwig University of Freiburg (livMatS) FIT‐Freiburg Center for Interactive Materials and Bioinspired Technologies Georges‐Köhler‐Allee 105 D‐79110 Freiburg Germany; ^2^ Albert Ludwig University of Freiburg Department of Microsystems Engineering (IMTEK) Georges‐Köhler‐Allee 102 D‐79110 Freiburg Germany

**Keywords:** adhesive composites, debonding‐on‐demand, mechanical compression, microcapsules, plasticizers

## Abstract

Temporary adhesives capable of forming strong yet easily reversible bonds are garnering significant interest as sustainable materials that facilitate the recycling and recovery of high‐value components. Herein is presented a comprehensive design and parameterization framework for developing effective temporary adhesives with mechanically induced debonding‐on‐demand capabilities. This framework is achieved by incorporating hexyl acetate‐filled microcapsules into commercial polyvinyl acetate adhesives, creating a responsive adhesive composite. Under controlled compression, these microcapsules rupture precisely, releasing their contents to induce sufficient adhesive plasticization to enable effortless debonding. Our results indicate that while the inclusion of microcapsules affects adhesion strength and toughness, the overall mechanical performance remains stable across different concentrations. Thermal tests highlight a 50 wt.% microcapsule concentration as optimal for enhanced plasticization, while compression tests show that an applied force of 5 kN achieves maximum capsule rupture without compromising substrate integrity. Ultimately, specimens bonded with the responsive composite under compression exhibit a striking 1200% increase in creep rates compared to those bonded with the neat adhesive, allowing for effective debonding‐on‐demand—an outcome unattainable with the neat adhesive. This simple and highly versatile approach lays the groundwork for advancing the development of more sustainable and functional adhesive materials.

## Graphical abstract

Debondable adhesives hold promise for advancing sustainable bonding solutions, yet their adoption is often constrained by synthetic challenges that limit practicality and scalability. This study demonstrates how encapsulated plasticizers can impart mechanically triggered debonding‐on‐demand functionality to commercial adhesives. By offering a straightforward, adaptable approach, it advances the development of more sustainable, versatile adhesives with enhanced debonding capabilities.

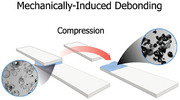

## 1 Introduction

With a global market exceeding €46 billion, the adhesive industry holds a crucial role in modern manufacturing, impacting major sectors such as automotive, aerospace, electronics, construction, healthcare, and consumer goods.^[^ ^1^, ^2^ ^]^ Adhesives enable efficient bonding of diverse substrates, enhancing product durability, aesthetics, and performance while reducing weight and allowing for flexible designs without visible fasteners.^[^ ^3^ ^]^ Still, traditional adhesives create permanent bonds that pose significant sustainability challenges by hindering disassembly and material reclamation efforts. These limitations lead to increased waste accumulation,^[^ ^4^ ^]^ often ending up in landfills or incineration, releasing CO_2_ and other greenhouse gases that exacerbate climate change. As a result, the recycling rate was only 11.7% in 2021, falling well short of the 23.4% target set for 2030.^[^ ^5^, ^6^ ^]^ As concerns over environmental impact and climate change grow, the adhesive industry faces increasing pressure to adopt more sustainable practices driven by regulatory changes, rising consumer awareness, and technological advancements.^[^ ^7^, ^8^, ^9^ ^]^ Innovations such as renewable materials, energy‐efficient manufacturing processes, and carbon capture technologies are transforming adhesive production to reduce environmental impact across their lifecycle.

In response to the increasing demand for sustainability, debondable adhesives that enable controlled disassembly of substrates without destructive methods has emerged as an interesting technology.^[^ ^10^, ^11^ ^]^ By integrating debonding mechanisms into adhesive formulations, manufacturers can improve the lifecycle sustainability of products, reducing waste and energy consumption compared to traditional disposal methods and ultimately promoting a circular economy by enabling efficient material reuse and recycling.^[^ ^12^, ^13^ ^]^ For instance, in the electronics industry, such as with smartphones, debonding‐on‐demand technology allows for easy disassembly of components at the end of a device's life. This facilitates efficient recycling of valuable materials and reduces electronic waste, promoting sustainability and resource conservation.^[^ ^14^ ^]^

Various approaches have been explored to create adhesives with debondable characteristics. For example, polymer matrices incorporating responsive motifs that dissociate and reassemble the matrix upon external stimulation, (i.e., heat, light, magnetic fields, or electrical signals) have been proposed.^[^ ^15^ ^]^ However, achieving consistently reliable and adjustable adhesion remains challenging due to the intricate nature of these systems. Not all external triggers can be universally applied to different materials and bonded areas without risking damage or altering the substrates. Moreover, synthesizing new adhesives typically involves complex formulation processes that require precise control over chemical reactions and parameters, potentially increasing costs and limiting scalability. As a result, these methods are often disregarded by industry due to their complexity and the challenges associated with achieving reliable and universally applicable adhesion across different materials and bonding scenarios.

In this context, the use of functional fillers that can be easily integrated into a matrix to achieve controllable adhesive properties on demand has gained more attention, as these fillers can leverage existing technologies and processes, enhancing the accessibility and implementation of advanced adhesives across various industries.^[^ ^16^ ^]^

One popular industrialized approach uses thermoexpandable microspheres,^[^ ^17^ ^]^ which consist of a polymer shell encapsulating a gas or volatile liquid. When heated, these microspheres expand over 100 times their volume,^[^ ^18^ ^]^ exerting pressure on the adhesive matrix and causing it to debond. Sakurai and colleagues applied this method to dismantle epoxy‐bonded plywood boards using a composite with 30 wt.% of thermally expandable particles.^[^ ^18^ ^]^ Ishikawa et al. later improved this technique for bonding wallpaper to plywood or plasterboards in construction.^[^ ^13^, ^19^ ^]^ This technology has also proven useful for diverse recycling purposes and has been employed in car manufacturing to remove windscreens, saving time and clean‐up costs.^[^ ^18^, ^20^, ^21^ ^]^

Another effective framework involves the controlled release of debonding agents from microcapsules dispersed within an adhesive matrix. When triggered by an external stimulus, these microcapsules release their contents, leading to the controlled debonding of the adhesive. As an example, Lee et al. developed core–shell nanocapsules with a poly(methyl methacrylate) (PMMA) core and a polyethyleneimine shell, filled with methylcyclohexane. During thermal treatment, the vaporized core created bubbles at the adhesive interface, aiding in the detachment of the adhesive film.^[^ ^22^ ^]^ It has been shown that incorporating just 1 wt.% of nanocapsules reduces adhesive strength by 57.6% and that the adhesive strength could be fine‐tuned by adjusting the temperature, duration, and quenching conditions of the thermal process. Additionally, further heating could be restored with further thermal treatment, as the capsules do not expand.

While thermoactivable particles have been well‐studied and widely used, other activation schemes for triggering debonding remain rare. This scarcity is quite surprising, considering that various stimuli, (i.e., mechanical forces,^[^ ^23^ ^]^ chemical (redox, pH),^[^ ^24^, ^25^ ^]^ electrical current,^[^ ^26^ ^]^ and light^[^ ^27^ ^]^) have been employed to activate nano/micro‐containers in polymer materials to trigger functions such as drug delivery,^[^ ^28^ ^]^ fragrance release,^[^ ^29^ ^]^ damage‐sensing,^[^ ^30^, ^31^ ^]^ and self‐healing.^[^ ^23^ ^]^ In the context of adhesive materials, such^[^ ^28^ ^]^ microencapsulation technology has so far only been used to separate reactive components, ensuring precise curing just before application, as demonstrated by commercial products from 3M.^[^ ^32^ ^]^

**Figure 1:**
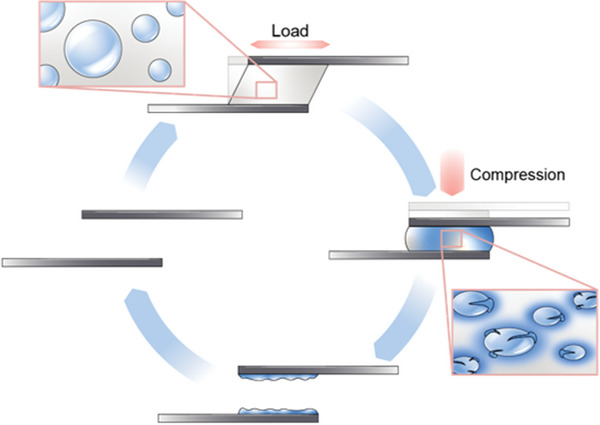
Illustration demonstrating the concept of mechanically‐induced debonding‐on‐demand. This approach involves an adhesive composite containing microcapsules (MCs) filled with a solvent, which bonds two substrates together. When mechanical compression is applied, the capsules rupture, releasing the solvent, which acts as a plasticizer. The plasticization increases malleability of the adhesive, allowing the substrates to separate and be reused for further life cycle applications.

We herein expand capsule‐based adhesive technology with an innovative mechanical activation scheme for debonding. Our framework uses controlled mechanical pressure to release a chemical solution from poly(urea‐formaldehyde) (PUF) microcapsules, plasticizing the adhesive matrix. This solvent diffusion enhances the matrix's malleability, allowing for on‐demand disassembly and reuse of bonded substrates when subjected to loading (**Figure** **1**). Unlike complex, synthetically demanding debonding methods, our approach integrates responsive microcapsules into commercial adhesives, such as polyvinyl acetate, through simple mixing, effectively bonding, and holding PMMA substrates. The debonding function is triggered by high compression forces, offering a practical and efficient solution. This method enables precise control over the intensity and duration of applied force, a crucial aspect when working with delicate materials. Additionally, mechanical activation is simpler and safer than approaches like chemical, UV, or temperature triggers, which involve complex processes and risks. Our study identifies key components for an effective system, including adhesive debonding mechanism analysis, microcapsule‐adhesive compatibility, adhesive formulation, bonding process, and mechanical activation, all of which ensure capsule integrity while optimizing debonding‐on‐demand. This model can, *à priori*, be adapted to other adhesives and material systems, providing a versatile platform for future innovations.

## 2 Discussion

Among various commercial adhesives, polyvinyl acetate was selected as the model gluing system to demonstrate the envisioned framework owing to its widespread availability and practicality (Figure S1a, Supporting Information). PVAc is commonly used as an all‐purpose/universal adhesive for physically bonding a variety of surfaces, including glass, wood, ceramics, and metal.^[^ ^33^ ^]^ Additionally, the polarity of PVAc allows it to dissolve in a range of organic solvents with moderate polarity, particularly those containing ester, ketone, or alcohol functional groups.^[^ ^34^, ^35^ ^]^ In parallel, hexyl acetate was chosen as plasticizer for PVAc, primarily due to their suitable chemical compatibility. Both PVAc and hexyl acetate contain ester groups, allowing them to interact effectively through dipole–dipole interactions, thus enhancing solubility.^[^ ^35^ ^]^ Additionally, hexyl acetate's favorable boiling point (bp = 164–176 °C, P(20 °C) = 0.16 kPa)^[^ ^36^, ^37^ ^]^ and melting temperature (−80 °C) have previously enabled successful microcapsule encapsulation processes, allowing controlled mechanical release of payloads.^[^ ^14^, ^30^, ^38^, ^39^ ^]^

To demonstrate the plasticization of PVAc adhesive, a physically cured PVAc film (22 mm long, 6 mm wide, and 0.6 mm thick) was immersed in 12.6 mL of pure hexyl acetate. Within minutes, the self‐supporting adhesive film (Figure S1b, Supporting Information) began to visibly soften and dissolve as the solvent infiltrated the matrix (Figure S1c, Supporting Information). Complete dissolution of the film was observed after 20 min, confirming the effectiveness of hexyl acetate as a plasticizer for debonding purposes (Figure S1b,c, Supporting Information).

Following reported procedures, hexyl acetate was introduced into double‐walled PUF microcapsules (MCs), through an emulsion polycondensation reaction involving urea, resorcinol, and formaldehyde.^[^ ^14^, ^31^ ^]^ The encapsulation process was carried out at 55 °C, with 1‐octanol and ethylene maleic anhydride copolymer serving as surfactant and dispersing aid, respectively (see supporting information). Note that double‐walled PUF containers were specifically chosen for their reliability in producing capsules that remain intact during the processing of composites with host polymers, rupturing only under applied mechanical force. This design also ensures improved long‐term payload isolation at room temperature and superior thermal stability compared to single‐walled capsules.^[^ ^14^, ^30^, ^31^ ^]^ Optical microscopy analysis of the resulting MCs revealed spherical‐shaped particles with an average diameter of 39 ± 16 µm (Figure 1a), and a size distribution ranging from 10 to 80 µm (Figure S2a, Supporting Information). It is noteworthy that the micrographs showed that most of the MCs remained intact after washing and purification processes (Figure S2b, Supporting Information). Further analysis with scanning electron microscopy determined an MC shell thickness ranging between 0.9 and 1.1 µm (Figure S3, Supporting Information). Additionally, thermogravimetric analysis revealed MCs’ thermal stability up to 110 °C, with a gradual mass loss of 10% occurring between 110 and 200 °C, typically attributed to the evaporation of hexyl acetate and possibly occurring through the capsule walls (Figure S4, Supporting Information).^[^ ^30^ ^]^ Consistent with previous reports, a significant mass loss of ≈96% was observed beyond this temperature threshold, likely due to capsule rupture and complete solvent evaporation.^[^ ^14^, ^31^ ^]^

To determine an ideal composite adhesive formulation for effective debonding capabilities and to evaluate processing feasibility while preserving capsule integrity, hexyl acetate MCs were incorporated into PVAc at concentrations ranging from 0 to 50 wt.% (processing specifics are provided in the Supporting Information). Note that the thickness of adhesives used in practical applications can vary based on specific needs and requirements. However, in several conventional adhesive applications, such as bonding surfaces in manufacturing or assembly processes, the adhesive thickness often ranges from a few micrometers to several hundred micrometers, with common values falling within the range of 100 to 500 µm.^[^ ^40^, ^41^, ^42^, ^43^ ^]^ Therefore, MCs‐based composite with thicknesses of 250–500 µm were prepared for further experimentation.

**Figure 2:**
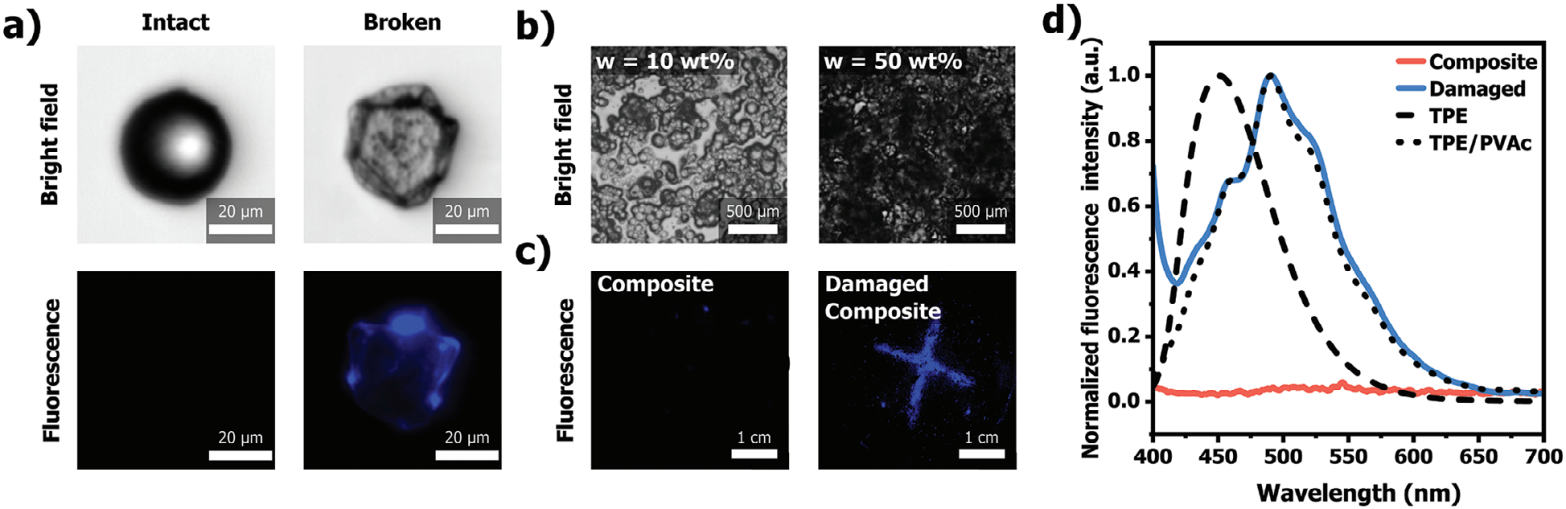
a) Top left: a micrograph of a single intact microcapsule; top right: a micrograph of a compressed microcapsule after one day. The bottom images show the corresponding fluorescence micrographs. b) Micrographs of PVAc/MCs composite loaded with 10 and 50 wt.% MCs. c) Fluorescence micrographs of intact (left image) and damaged (right image) PVAc/MCs composites embedded with 50 wt.% MCs filled with a solution of TPE in hexyl acetate (*c* = 0.02 m). The incision in the composite material was made using a razor blade. d) Comparison of fluorescence spectra of the PVAc/MCs composite before (red) and after incision (blue) (*F* = 2.5 kN for *A* = 1 cm^2^). Neat crystalline TPE (dashed line) and blended TPE in PVAc (dotted line) serve as references. PVAc/TPE film was cast from a mixture of PVAc adhesive and TPE in a hexyl acetate solution. All fluorescence micrographs and solution measurements were recorded under UV excitation at *λ*_ex_ = 365 nm.

Micrographs of the PVAc/MCs composite films demonstrated a moderately uniform dispersion at ratios of 10%, 20%, and 30% (**Figure** **2**b; Figure S5, Supporting Information). However, assessing the integrity of microcapsules in composites containing more than 10 wt.% MCs proved to be challenging (Figure 2b; Figure S5, Supporting Information). Given that the effectiveness of the targeted debonding depends on the number of capsules ruptured upon the application of mechanical force, accurate control over their structural integrity is crucial. To achieve a precise characterization, self‐reporting systems utilizing established luminescent probes were employed to evaluate capsule integrity.^[^ ^31^ ^]^ Thus, 1,1,2,2‐tetraphenylethylene (TPE), a commercially available luminogen that induces fluorescence upon aggregation, was mixed with hexyl acetate (*c* = 0.02 m). When encapsulated using the previous procedure (see supporting information), the TPE chromophore remains non‐emissive, resulting in colorless microcapsules under UV irradiation (Figure 2a, left). Note that this colorlessness is also attributed to the core material's transparency to visible light.^[^ ^31^ ^]^ However, upon damage and release of the solution payload into the matrix, the solvent evaporates, causing the chromophore to aggregate and produce a blue fluorescence, at *λ*_ex_ = 365 nm (Figure 2a right). This resulting turn‐on colorimetric effect was used as a readout signal to assess capsule integrity during composite processing. Gratifyingly, the incorporation of these TPE/MCs into the composite formulations effectively demonstrated the absence of emission in all prepared composites up to 50 wt.%, establishing this ratio as the maximum processing limit for the composite. In contrast, composites with intentionally damaged microcapsules displayed blue fluorescent spots in the micrographs (Figure 2c), thereby validating the use of such a self‐reporting approach to confirm the integrity of composites with high capsule concentrations (>10 wt.%).

**Figure 3:**
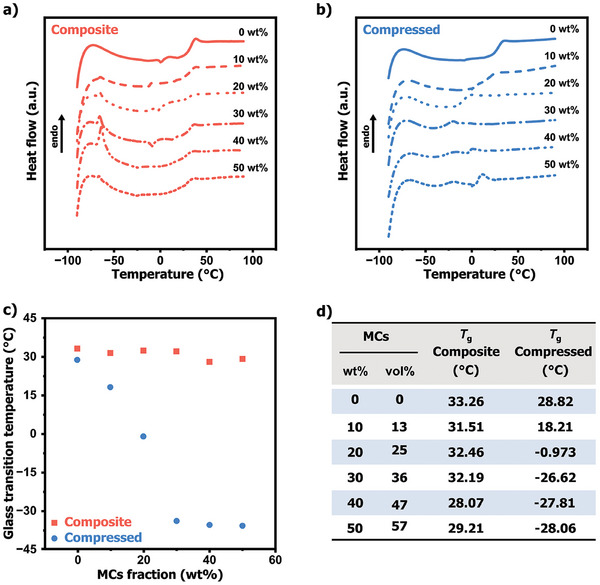
Differential scanning calorimetry (DSC) traces of PVAc/MCs composites embedded with different MCs weight fractions, from 0 to 50 wt.% a) before and b) after compression with a force of 1 kN over an area of 1 cm × 1 cm. c) Plot of the corresponding glass transition temperatures (*T*_g_), measured in (a,b), as a function of the MC weight fraction. d) Table presenting the *T*_g_ values of neat PVAc film and PVAc/MCs composite (with capsule loadings ranging from 10 to 50 wt.% and their corresponding volume fraction) both before (referred to as “intact”) and after 5 kN compression (referred to as “compressed”). Films were prepared with a thickness of ≈500 µm and an area of ≈1 cm^2^. All DSC traces were recorded at heating and cooling rates of 10 °C min⁻¹ and are presented with vertical superimposition.

After establishing a suitable composite processing method, the mechanical release of hexyl acetate and its subsequent plasticizing effect on PVAc were evaluated using differential scanning calorimetry (DSC). Experiments were conducted on the different as‐prepared composite films containing 10–50 wt.% MCs, as well as on comparable samples that had been freshly compressed to a force of 50 MPa (corresponding to a load of 5 kN for a film of dimensions of 1 cm × 1 cm × 500 µm). The force was chosen based on earlier reports indicating that capsules rupture under forces greater than 50 N applied to an area of 1 cm^2^.^[^ ^30^ ^]^ Thus, applying a force above this threshold is expected to guarantee complete particle breakage. In real‐life adhesive applications, such pressures are comparable to those applied during manual assembly or using clamps in industrial settings to ensure uniform bonding and alignment.^[^ ^44^ ^]^ The success of this compression process was verified and confirmed by the appearance of a blue fluorescence (Figure S6, Supporting Information). Note that these samples were compressed immediately before performing the DSC measurements (see supporting information). All as‐prepared materials exhibited a glass transition temperature (*T*_g_) value ranging between 28 and 32 °C, slightly lower than the neat PVAc, typically displaying a *T*_g_ of 33 °C (**Figure** **3**a,c,d).^[^ ^34^, ^35^ ^]^

It is worth noting that slight variations in the *T*_g_ values observed in other reports may result from differences in the material processing techniques. Comparatively, the DSC traces of the compressed composites revealed a substantial decrease in *T*_g_ up 50 °C, in conjunction with the increasing concentration of MCs in the composite (Figure 3b,c,d). Note that at MC contents exceeding 40 wt.%, a second glass transition, occurring ≈7 °C, becomes apparent, with its area expanding as the MC fraction increases. This transition is possibly attributed to the separation of hexyl acetate into distinct phases within the material, each with its own glass transition temperature (*T_g_*). Overall, all composite formulations, clearly demonstrate the potential of MCs to activate the plasticizing effect under compression. Additionally, the decrease in *T*_g_ below ambient temperature suggests a potential enhancement in the adhesive's malleability, which would ultimately favor the targeted debonding function.

## 3 Adhesive Properties

**Figure 4:**
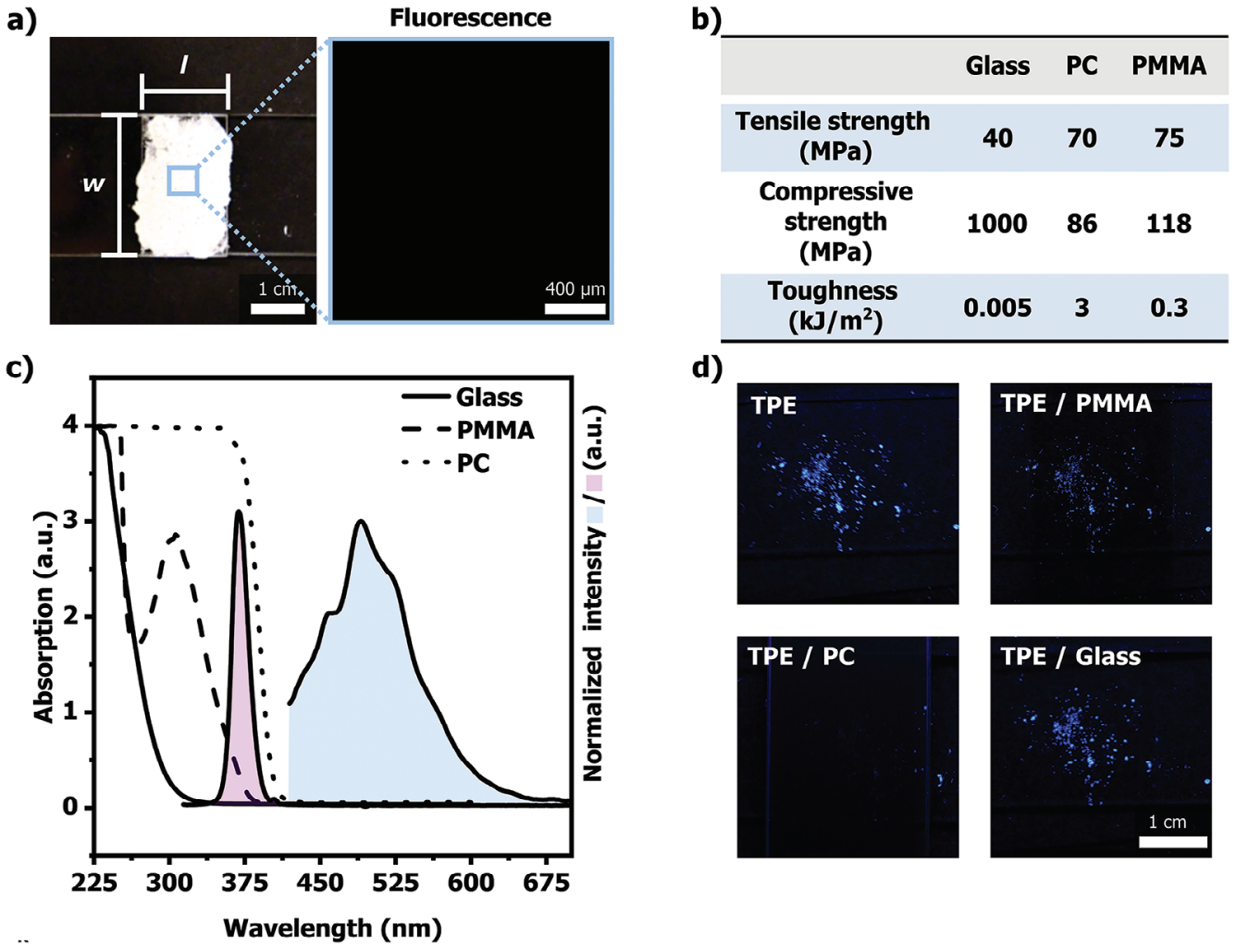
a) Lap joint specimen bonding two PMMA substrates using the PVAc/MCs composite (MC: 50 wt.%) over an area measuring 25 mm wide × 15 mm length. The insert shows a fluorescence micrograph taken on the bonded area at *λ*_ex_ = 365 nm. b) Table of mechanical properties for the different substrates, including tensile strength, compressive strength, and toughness.^[^ ^42^, ^43^ ^]^ c) UV–vis absorption spectra of glass, PMMA, and PC substrates measured in the solid state. For reference, the fluorescence spectrum of a damaged composite film (MCs: 50 wt.%) is shown in blue, while the emission spectrum of the UV lamp used for excitation is shown in purple (*λ*_ex_ = 365 nm). d) Photographs of neat crystalline TPE and TPE covered with glass, PMMA, and PC substrates, captured under a UV lamp at *λ*_ex_ = 365 nm.

While DSC measurements indicate that capsule rupture causes mechanically‐induced plasticization of PVAc, the optimal composite composition for achieving effective debonding in bonded substrates remains still uncertain. To evaluate the performance of the produced capsule‐based adhesive composites as a mechanically‐debondable system, single lap joint specimens were prepared. Films of various composites (ranging from 10 to 50 wt.%) were sandwiched between two PMMA substrates, with an overlap area measuring 15 mm in length and 25 mm in width (**Figure** **4**a). PMMA slides were chosen as substrate material on account of their transparency in the UV regime, allowing for the detection of aggregated TPE absorption (excitation: *λ*_max_ = 365 nm, emission: *λ*_max_ = 495 nm) (Figure 4c). This feature enables the monitoring of microcapsule integrity during the composite processing as well as during the gluing procedure. When compared to other potential transparent substrate materials that could also serve monitoring purposes, that is, glass (transmittance at *λ*_max_ > 300 nm) or polycarbonate (PC) (transmittance at *λ*_max_ > 410 nm) (Figure 4c,d), PMMA additionally offers the advantage of robust mechanical stability with a high elastic modulus (*E* = 0.7 GPa),^[^ ^45^ ^]^ necessary to withstand mechanical testing with PVAc composites in the later stages of this research. It is worth noting that, while PC has good mechanical properties (*E* = 1.18 GPa), its optical properties are comparatively less favorable (Figure 4b–d; Figure S7a, Supporting Information).^[^ ^45^ ^]^ On the other hand, while glass has suitable optical properties, it is too brittle (*E* = 60 to 100 GPa)^[^ ^46^ ^]^ to endure the intended mechanical loading in this case (Figure 4b; Figure S7b, Supporting Information).

**Figure 5:**
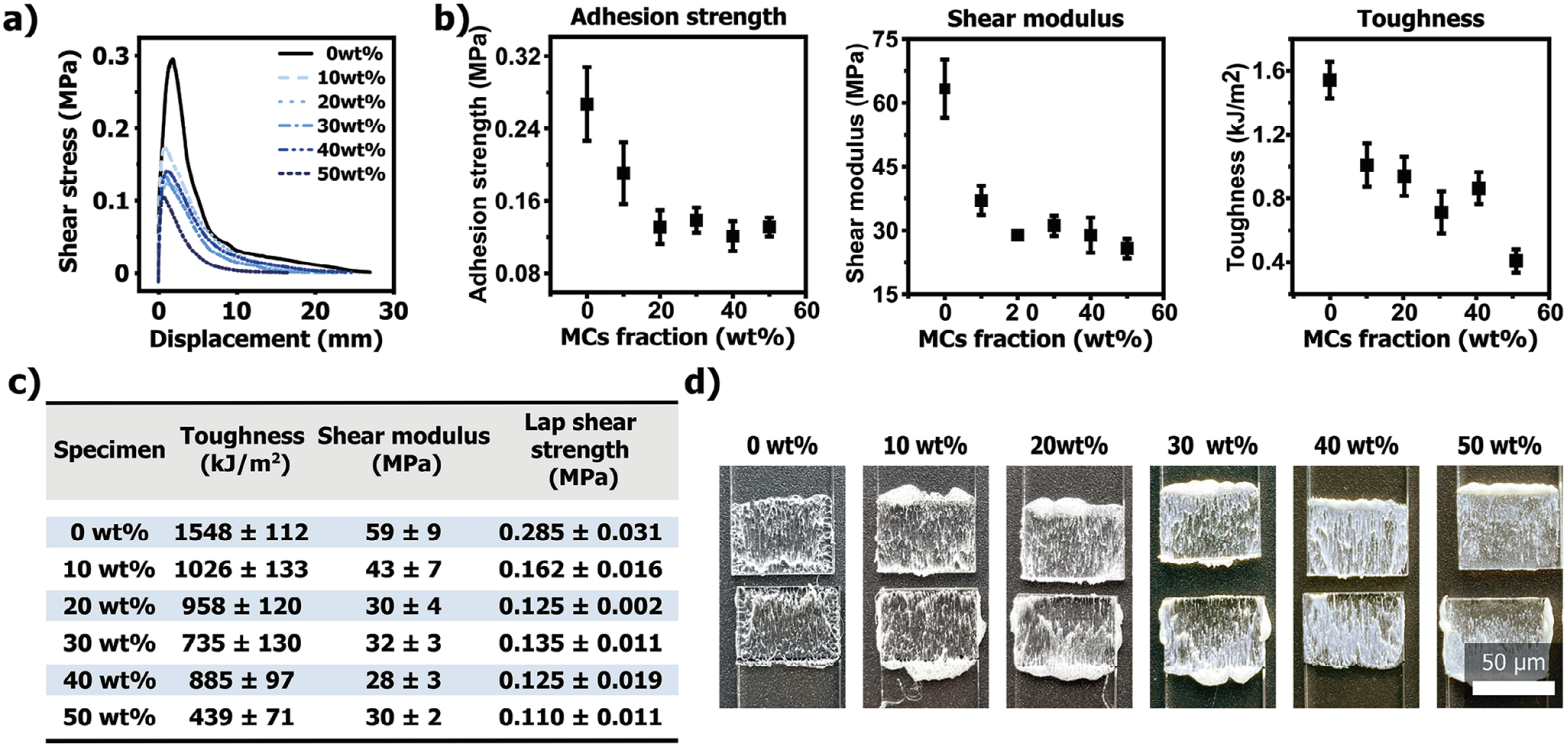
a) Displacement curves for neat PVAc and PVAc/MCs composites (10 to 50 wt.%) measured during lap‐shear tests. b) Plots depicting the toughness, shear modulus, and adhesion strength as a function of MCs weight fraction. c) Table with the corresponding values illustrated in (b). d) Photographs of the cohesive failure of PVAc and the various PVAc/MCs composites.

On this basis, specimens built with PMMA were glued together and left to dry under ambient conditions for 6 days to ensure proper adhesion.^[^ ^33^ ^]^ Encouragingly, fluorescence optical micrographs taken at the joint surface revealed an absence of fluorescence, indicating the integrity of the capsules and the robustness of the sample preparation process (Figure 4a; Figure S8a, more details available in the Supporting Information). Following specimen preparation, lap‐shear tests were conducted to quantitatively evaluate the adhesive strength of all composite formulations. The tensile lap‐shear measurements of the specimens revealed that both the neat adhesive and the composites exhibit typical stress shear behavior observed for uncrosslinked adhesives (**Figure** **5**a; Figure S9, Supporting Information). These curves typically show an increase in shear stress (0–0.3 MPa) as displacement increases, until the shear stress reaches a maximum (0.110 to 0.285 MPa), known as the adhesion strength (Figure 5c, see calculation specifics in supporting information). This failure point is then followed by a progressive loss of resistance properties attributed to the loss of mechanical integrity in the adhesive. The lap‐shear strength was determined from the load–displacement curves by dividing the failure load by the lap joint area.^[^ ^47^ ^]^ Composite specimens revealed a loss of adhesion strength ranging from 50% to 62% when compared to the neat PVAc adhesive (Figure 5b,c). Although the strength loss was substantial (40%) for the 10 wt.% composite, similar strength losses (55% to 59%) were observed for higher composite concentrations (20, 30, 40, and 50 wt.%) (Figure 5b, left).

While it can be argued that the incorporation of particles may alter the surface adhesion and consequently the resulting adhesion strength,^[^ ^48^ ^]^ observations of the specimens after failure revealed that the adhesives failed cohesively—suggesting the failure occurred within the adhesive layer itself rather than at the interface between the adhesive and the substrate (Figure 5d). Additionally, X‐ray microtomography further supports this observation, showing no surface enrichment of the capsules (Figure S10, Supporting Information). Previous studies have suggested that the reduction in adhesion strength can be attributed to higher stress concentrations at the matrix‐(hollow) microsphere interfaces, leading to debonding between the matrix and microspheres.^[^ ^49^ ^]^ Furthermore, it has been reported that increasing filler content in polymeric composites (from 30 to 60 wt.%) correlates with a reduction in adhesion strength, resulting in a decrease from 30 to 10 MPa, as well as a decline in elongation at break.^[^ ^50^ ^]^ As anticipated, both the shear modulus, which indicates the material's stiffness under shear forces, and the toughness, which measures the material's ability to dissipate energy and deform plastically without fracturing, exhibited similar decreasing trends across all the composites compared to the neat PVAc adhesive.

Additional lap‐shear experiments were conducted at varying displacements to assess the integrity of the microcapsules under shear force (Figure S11a, Supporting Information). Photographs of the bonding area were taken under UV‐light illumination (at λ_ex_ = 365 nm) and subjected to red–green–blue (RGB) color analysis. RGB channel intensities were averaged to derive a mean grayscale value for the bonding area, which was plotted as a function of displacement (Figure S11b, Supporting Information). Note that this color analysis previously validated as a reliable method for detecting and quantifying optical responses in similar capsule‐based composites,^[^ ^30^ ^]^ demonstrated a minimum detection threshold at a 5 wt.% microcapsule concentration for the present system (Figure S12, Supporting Information). The photograph analysis of the stretched specimens revealed minimal microcapsule rupture within the displacement range of 0.1 to 0.6 mm, as indicated by low grayscale values (Figure S11b, Supporting Information). Specifically, both the shear modulus (measured between 0 and 0.01 mm) and shear strength (reached at ≈0.36 mm) remain unaffected, as their values are similar to those of the reference specimen before stretching. These results suggest that the observed changes in mechanical properties primarily arise from the incorporation of microcapsules (MCs), which likely disrupt the matrix structure and introduce stress concentrators at the capsule–matrix interfaces. As a result, the composite material becomes less effective at withstanding mechanical stresses, making it more prone to failure under load. While this structural disruption may contribute to the reduction in the composite's toughness and elongation at break,^[^ ^51^, ^52^ ^]^ it is difficult to isolate the effect of plasticization caused by capsule rupture during the test. In fact, the shear experiment, conducted to a displacement of 2.5 mm, exhibited fluorescence, suggesting that a significant number of capsules have ruptured (Figure S11b, Supporting Information). This observation further suggests that the release of hexyl acetate at higher displacements influences the material's mechanical response.^[^ ^51^, ^52^ ^]^

These results decisively demonstrate that, despite a reduction in adhesion properties, the composite achieves a reliable holding strength of 0.162 to 0.11 MPa (compared to 0.285 MPa for the neat adhesive) while preserving capsule integrity. This resilience ensures its suitability for a range of light to medium household applications, including repairing minor objects, fixing furniture, assembling DIY projects, and attaching larger fixtures—tasks typically requiring stresses within the sub‐MPa range. Moreover, this performance highlights the practical utility of the debonding‐on‐demand system, offering a versatile and reliable solution for modern adhesive applications.

## 4 Mechanically‐Induced Debonding‐on‐Demand

**Figure 6:**
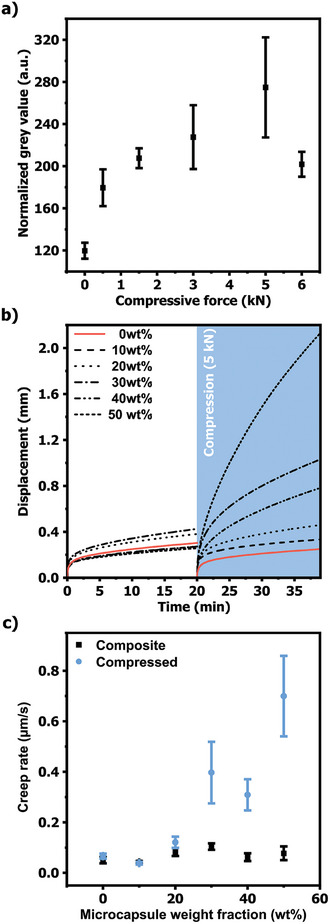
a) Plot of the normalized grey values, extracted from RGB analysis of images captured of the composite loaded with 50 wt.% MCs. b) Plot of the lap joint displacement as a function of the time obtained from creep experiments using neat PVAc and PVAc/MCs composites of different MCs weight fractions. The blue area depicts the creep behavior of the same samples after undergoing a 20‐minute creep experiment and then being subjected to a compressive force of 5 kN for <10 s. c) Plot of the creep rates as a function of the MCs weight fraction before and after compression by a force of 5 kN.

To evaluate the mechanically‐induced debonding capability of the composite adhesives, the compression force required to activate this function was investigated. Accurately determining this compression level is critical for ensuring effective debonding‐on‐demand while preserving the integrity of the substrate. Insufficient compression may not rupture enough capsules to release the solvent required for matrix plasticization, hindering effective debonding. On the other hand, excessive compression can displace the composite from the joint and damage the substrates, rather than promoting debonding. In this context, the self‐reporting functionality was employed once again to monitor the efficiency of the release process under compression. Specimens loaded with 50 wt.% MCs were subjected to compressive forces ranging from 0.5 to 6 kN and the resulting fluorescence emission was measured in the solid state after one week. This waiting period allowed for complete solvent evaporation, ensuring full aggregation of the TPE and accurate quantitative self‐reporting. Kinetics of the evaporation revealed that the conversion process occurred over 36 h (Figure S13, Supporting Information). The 50 wt.% formulation was selected due to its ability to maximize the plasticizing effect (DSC measurements, Figure 3) and maintain adhesion strength despite the high filler content (Figure 5a–c). Photographs of the bonding area were captured under UV‐light illumination (λ_ex_ = 365 nm) and the grey values for the bonding area, obtained through color analysis, were plotted as a function of the compressive force (**Figure** **6**a). Note that the color data were normalized by the adhesive bond thickness to account for the thinning effect caused by compression (more details on supporting information). The plots revealed a gradual increase in colorimetric intensity with the application of compressive forces ranging from 0 to 6 kN on the specimen, eventually reaching a saturation threshold beyond this range (Figure 6a). Consequently, 5 kN was identified as the optimal compression force to maximize capsule damage, thereby enhancing adhesive plasticization and debonding.

Accordingly, uniaxial creep tests were conducted on the specimens before and after compression (at 5 kN, corresponding to a stress of ≈13 MPa) to assess the mechanically‐induced debonding functionality for all composites with MCs weight fractions ranging from 10 wt.% to 50 wt.%. All specimens were subjected to a constant tensile load of 2.5 N at room temperature and their time‐dependent displacement was recorded over the course of 20 min. All composite specimens exhibited similar creep strain profiles, with time‐dependent variations comparable to those of the neat adhesive, suggesting that neither the addition of particles nor their concentration impact the mechanical performance of the neat adhesive under the applied force. These same specimens were then compressed at 5 kN with a strain rate of 10% min^−1^ (equivalent to <10 s) at room temperature, and their creep behavior subsequently measured. Gratifyingly, all composites displayed significant changes in their creep behavior and rates compared to the neat adhesive (Figure 6b; Figure S14, Supporting Information).

These differences are most evident in the plots depicting the creep rates as a function of the MCs weight fraction (Figure 6c). At weight fractions below 30 wt.%, the minor differences in rate suggest that the released solvent is insufficient to reverse the physical curing process. However, at fractions above this threshold, particularly at 50%, a remarkable increase in creep behavior was observed (1200% increase in creep rate compared to neat PVAc), establishing this formulation as the most effective for enhancing debonding functionality in the PVAc adhesive and the designed framework. Additionally, further creep experiments of the PVAc/MC composite with a 50 wt.% loading, conducted over three days, showed no material rupture (Figure S15, Supporting Information). This result confirms the composite's stability under prolonged stress, demonstrating its suitability for applications that require reliable performance under sustained loading over extended periods. It is worth emphasizing that, as one might anticipate, an increase in the applied load could shift the threshold at which debonding is triggered, allowing for a dynamic adjustment of the capsule concentration. This adaptability further highlights the remarkable versatility of the system, offering a level of customization that is uniquely suited to meet the specific demands of a wide array of applications. To further demonstrate this versatility, the same system was successfully embedded into another widely used commercial polychloroprene‐based adhesive, where it exhibited identical, mechanically‐induced debonding‐on‐demand upon compression (Figure S16, Supporting Information), highlighting the framework's robustness across different adhesive formulations.

## 5 Conclusion

To conclude, a comprehensive method has been devised to engineer innovative adhesive composites with mechanically‐induced debonding capabilities. Through a rigorous series of experiments—including encapsulation processes, composite formulation, mechanical testing, and debonding functionality evaluation—the study confirms the efficacy of hexyl acetate microcapsules (MCs) as plasticizers for polyvinyl acetate (PVAc) adhesives. This investigation also demonstrates the controlled release of these plasticizers upon mechanical activation, bringing *T*_g_ below ambient temperature and thus facilitating the disassembly and recycling of bonded materials. Among the formulations tested, a 50 wt.% concentration of MCs was found to be optimal, resulting in a creep increase 24 times greater compared to other formulations. Although the adhesive strength of the composites is lower compared to neat PVAc, these composite adhesives still meet practical requirements for many household applications.

This pioneering framework for mechanically‐induced debonding‐on‐demand provides valuable insights into the design, formulation, and performance of mechanically debondable adhesive composites. The seamless integration of microcapsules as functional fillers into existing matrices, combined with their ability to store and release agents via mechanical triggers, highlights their versatility and effectiveness in leveraging and advancing current technologies. This development significantly expands the range of possible applications for advanced adhesives, increasing their accessibility and practicality across a variety of industries. Additionally, while this study primarily focused on capsule concentration, its successful demonstration opens up opportunities for future research on other critical factors, such as capsule size, morphology, shell materials, and payloads. Exploring these variables holds the promise to further optimize the performance and expanding the versatility of debondable adhesives. This innovative approach has the potential to shift adhesives from linear consumption models to key enablers of a circular economy, promoting the recovery, repair, and reuse of adhesive‐based products.

## Conflict of Interest

The authors declare no conflict of interest.

## Supporting information



Supporting Information

## Data Availability

The data that support the findings of this study are available from the corresponding author upon reasonable request.
