# Supplementary material for: Adhesives with Debonding‐On‐Demand Capability: Leveraging Responsive Microcapsules for Mechanically‐Induced Debonding
Source: Adv Mater. 2025 Mar 3;37(13):2414308. doi: 10.1002/adma.202414308 (PMC11962676; doi:10.1002/adma.202414308)
Supplement: Supplementary file 1 — Supporting Information [file ADMA-37-2414308-s001.docx]

DOI: 10.1002/((please add manuscript number))

**Article type: Communication**

Adhesives with Debonding-on-Demand Capability: Leveraging Responsive Microcapsules for Mechanically-Induced Debonding.

Claas-Hendrik Stamp^1^, Jana Stump^1^, Céline Calvino^1,2^*

^1^C.-H. Stamp, J. Stumpp, C. Calvino

Cluster of Excellence livMatS, University of Freiburg (livMatS)

FIT-Freiburg Center for Interactive Materials and Bioinspired Technologies

Georges-Köhler-Allee 105, D-79110 Freiburg, Germany
E-mail: celine.calvino@livmats.uni-freiburg.de

^2^ C. Calvino

University of Freiburg – Department of Microsystems Engineering (IMTEK)

Georges-Köhler-Allee 102, D-79110 Freiburg, Germany
E-mail: celine.calvino@livmats.uni-freiburg.de

–––––––––

1. Set value for Heading 9

Table of Contents:

[1 Supporting Data S-2](#_Toc177804260)

[2 Supporting Experimental Section S-11](#_Toc177804261)

[3 References S-19](#_Toc177804262)

# Supporting Data


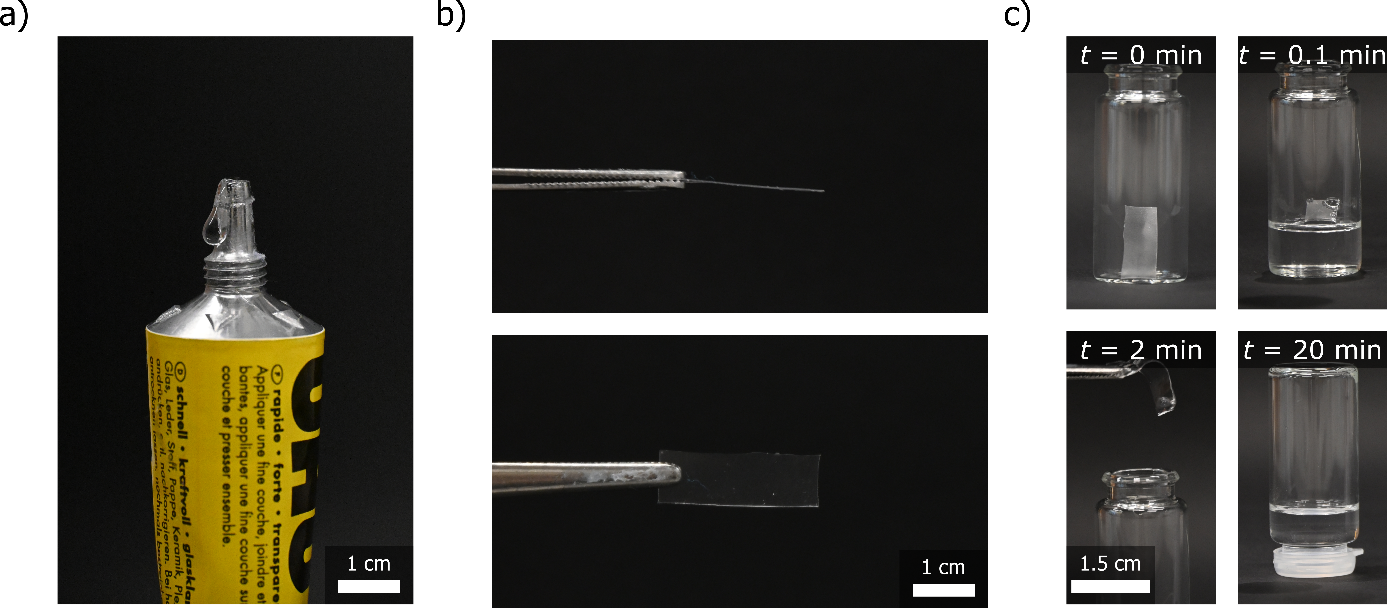


**Figure S1: a)** Commercially available, non-crosslinked polyvinyl acetate-based adhesive (PVAc). **b)** Self-supporting adhesive film after a curing time of t > 24 h under standard conditions (*i.e.* room temperature, relative humidity (RH) = 40 ‑ 60 %). **c)** PVAc film: top left shows the neat material, top right shows the film freshly immersed in hexyl acetate, bottom left shows the film after 2 minutes of immersion, and bottom right shows the completely dissolved PVAc after 20 minutes of immersion.


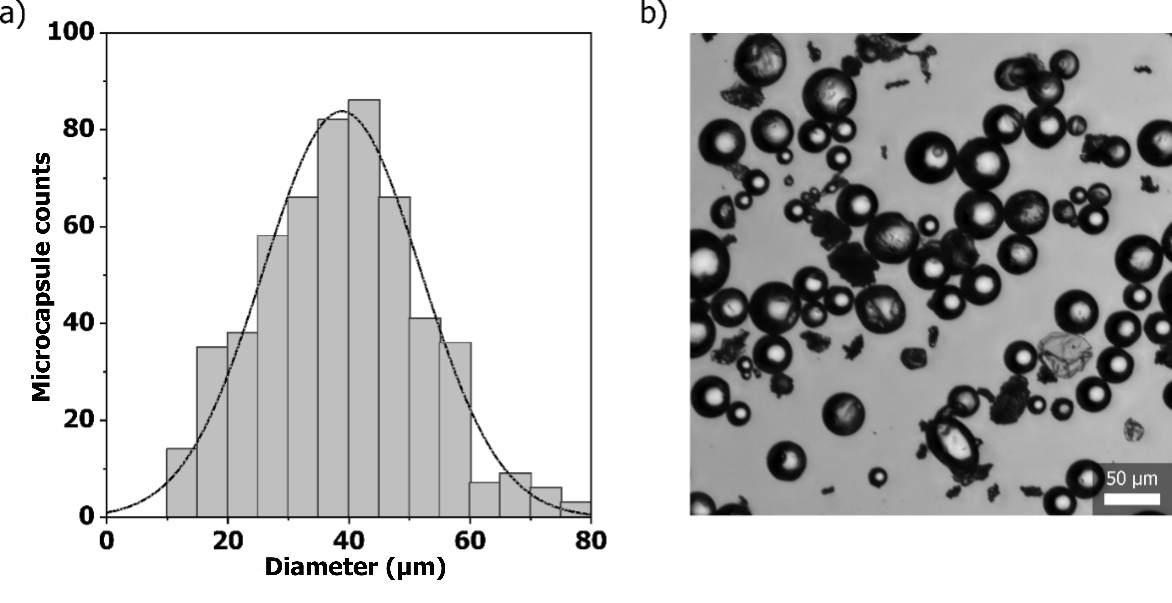


**Figure S2: a)** Histogram of the diameter distribution of microcapsules filled with 1,1,2,2-tetraphenylethylene (TPE) solution in hexyl acetate. **b)** Micrographs of the microcapsules (spherical objects) with isolated polymer fragments (irregular objects).


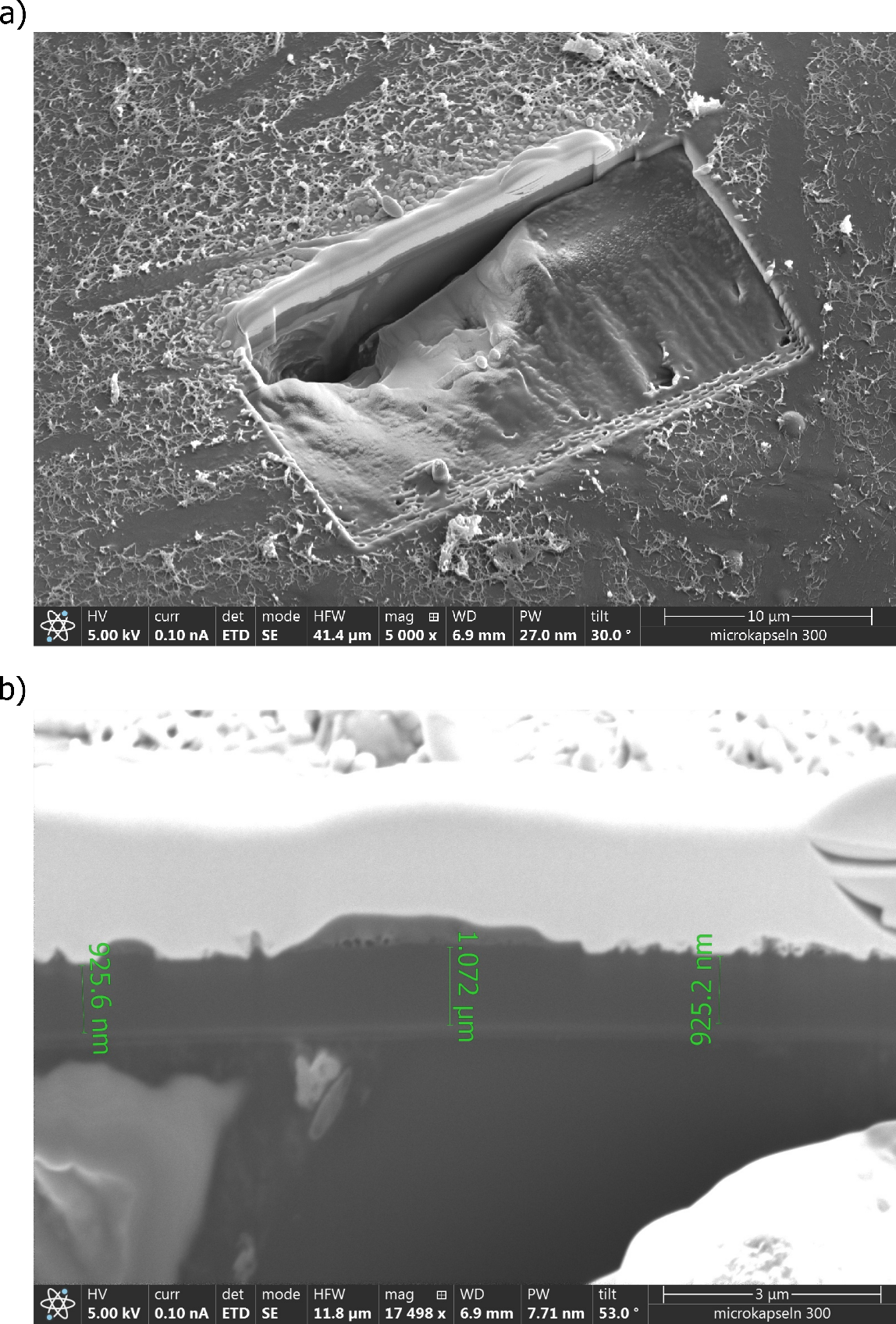


**Figure S3:** Scanning electron microscopy (SEM) of microcapsule shell made of polyurethane/poly(urea-formaldehyde). **a)** A rectangular cross-section of the microcapsule shell, cut using a focused ion beam (FIB). **b)** Zoom of the cross-section.


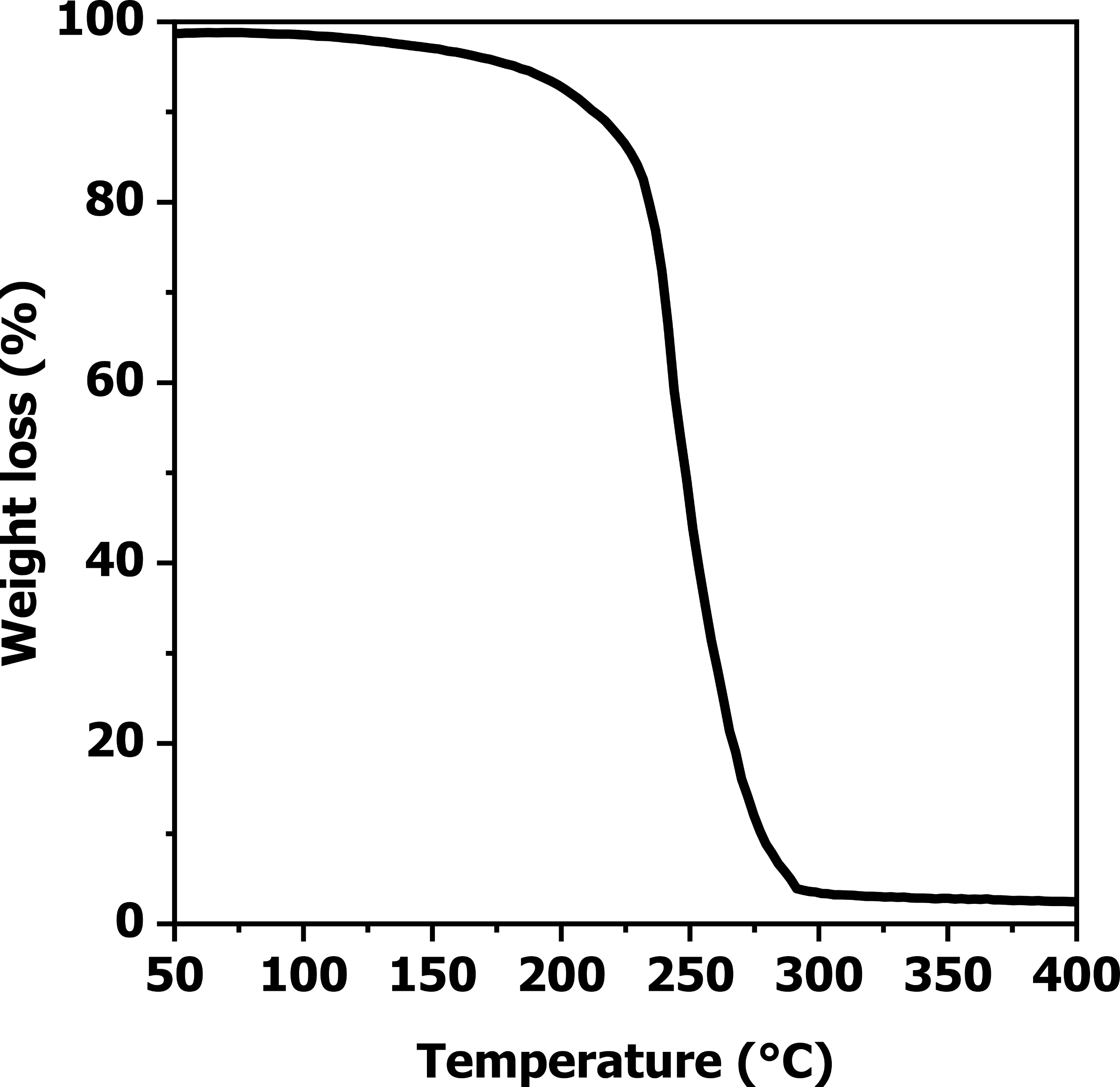


**Figure S4:** Thermogravimetric analysis (TGA) curve of the microcapsules filled with a solution of 1,1,2,2-tetraphenylethylene (TPE) in hexyl acetate. TGA experiments were carried out under N_2_ atmosphere with a rate of 5 °C min^-1^.


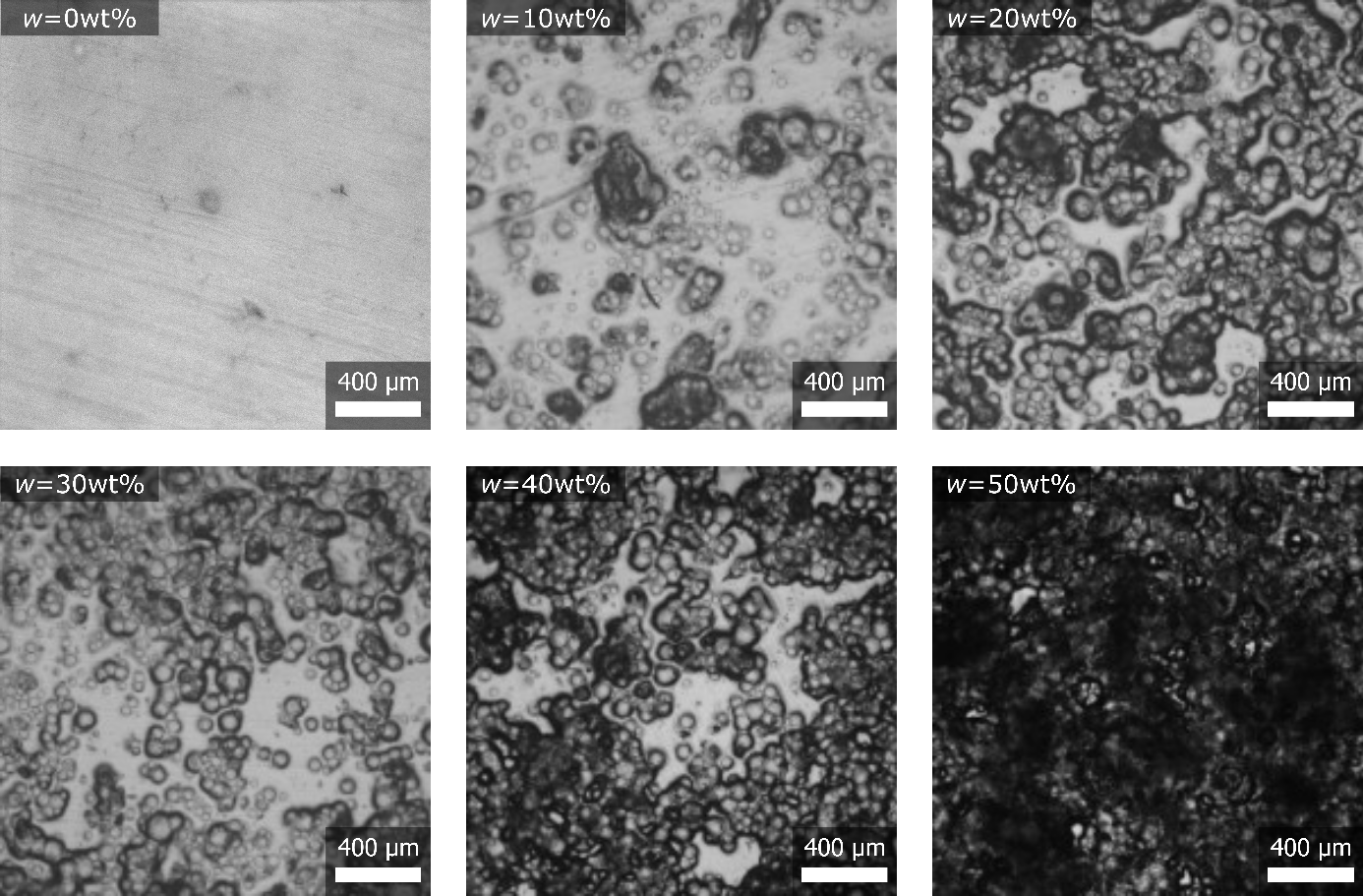


**Figure S5:** Optical micrographs of neat PVAc and PVAc/microcapsule composite films made of different microcapsule weight fractions, ranging from 10 to 50 wt %. All films were prepared with a thickness of ca. 600 µm.


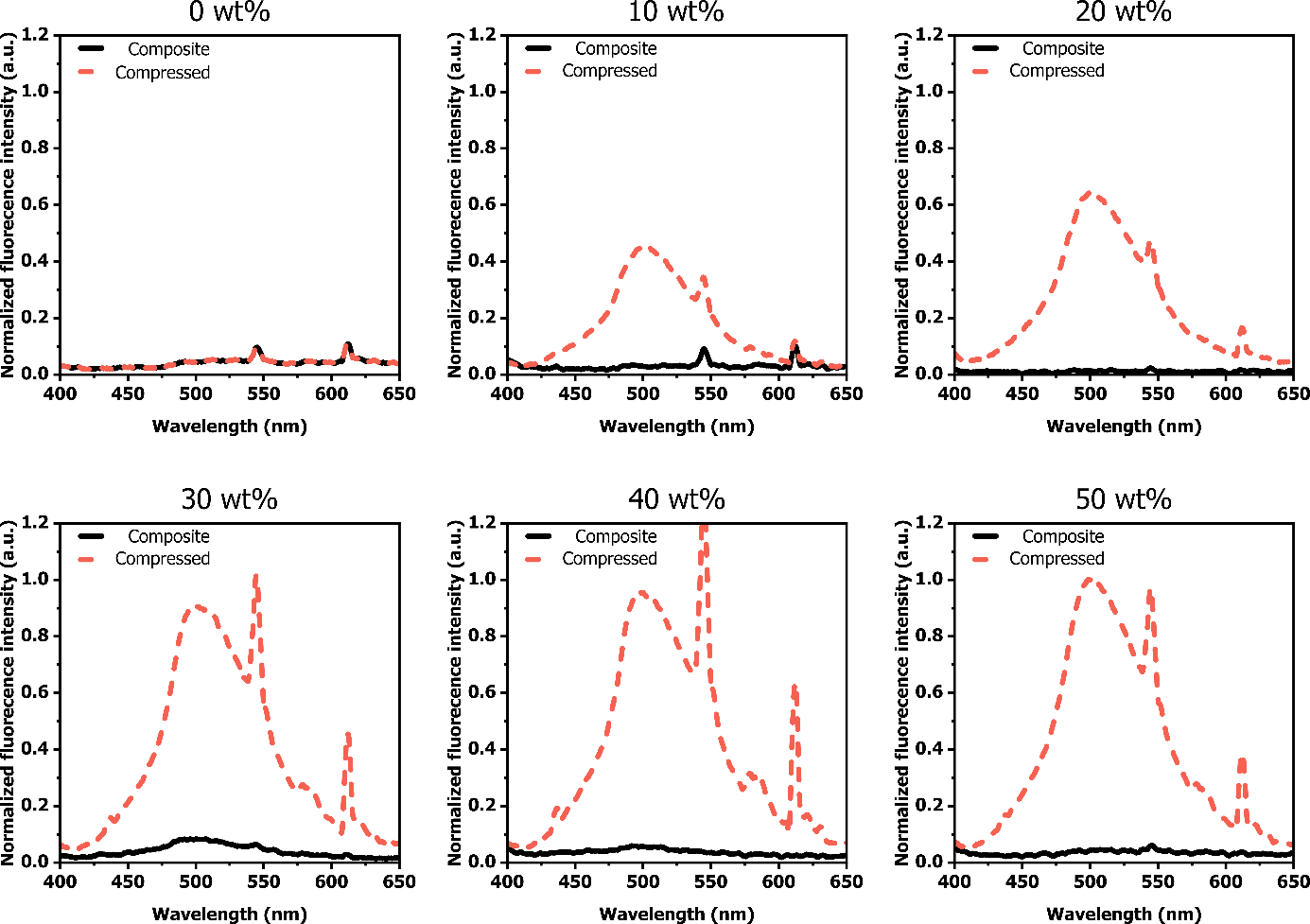


**Figure S6:** Comparison of the solid state fluorescence spectra (λ_ex_ = 365 nm) of neat PVAc film and PVAc/MCs composites (10-50 wt %) films before (referred as intact, black curves) and after 5 kN compression (referred as compressed, red curves). Films were prepared with a thickness of ca. 600 µm and an area of ca. 1 cm^2^.


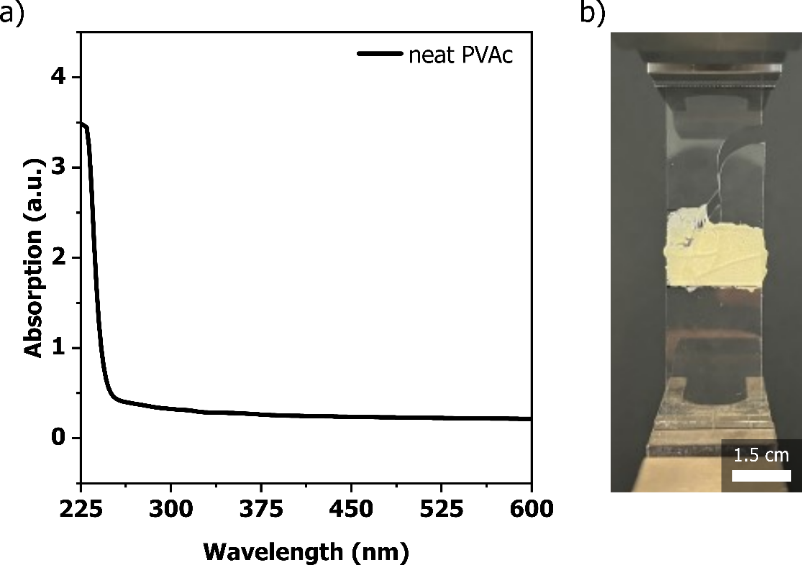


**Figure S7: a)** Solid state UV-vis spectroscopic measurement of neat PVAc film. The film was prepared with a thickness of ca. 500 µm and an area of ca. 1 cm^2^. **b)** Single-lap joint specimen bonding two glass substrates with 50 wt % PVAc/MCs composite, showcasing the failure of the glass substrate during lap adhesion strength testing.


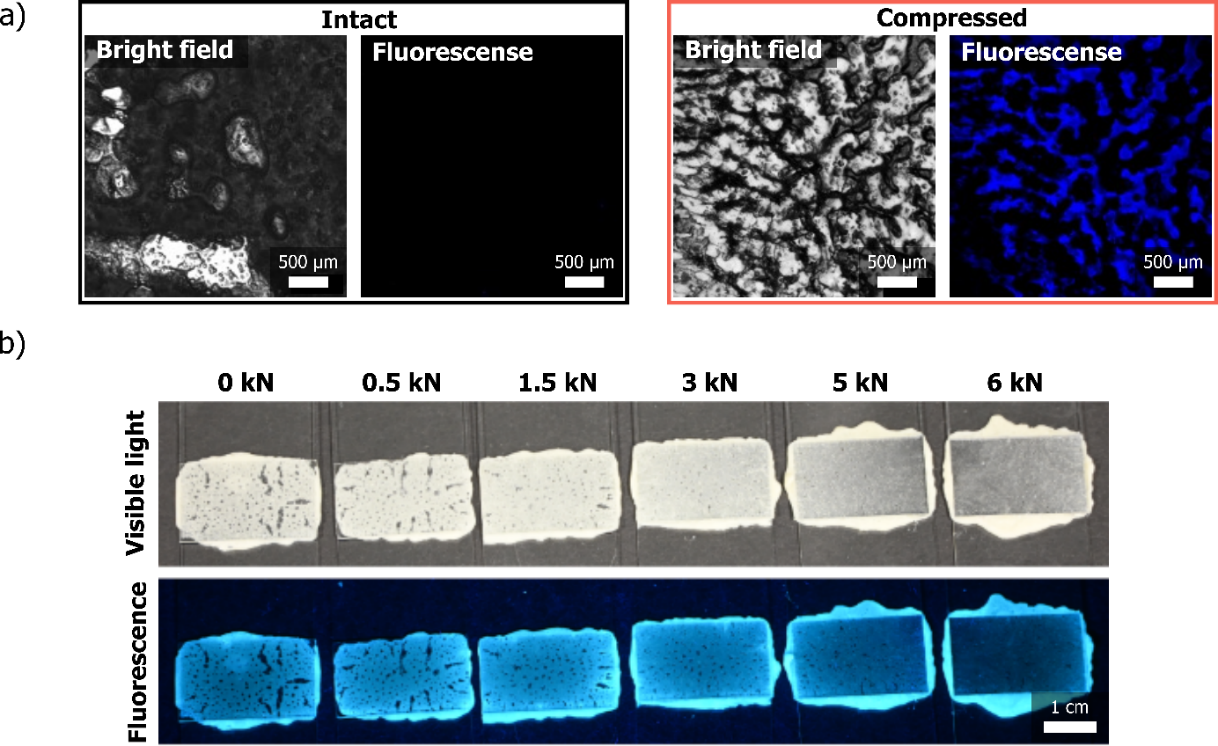


**Figure S8: a)** Micrographs of the bonded area of a single-lap joint specimen before (referred as intact) and after 5 kN compression (referred as compressed) under visible light (bright field) and upon irradiation at λ_ex_=368 nm. **b)** Photographs of the bonded area single-lap joint specimens after compression with forces ranging from 0 to 6 kN. The pictures shown were recorded under visible light, and UV illumination (λ_ex_ = 365 nm). All specimens in (a-b) were made of two glass substrates bonded with 50 wt % PVAc/microcapsule composite. The bonding area is 25 mm (width) x 15 mm (length).


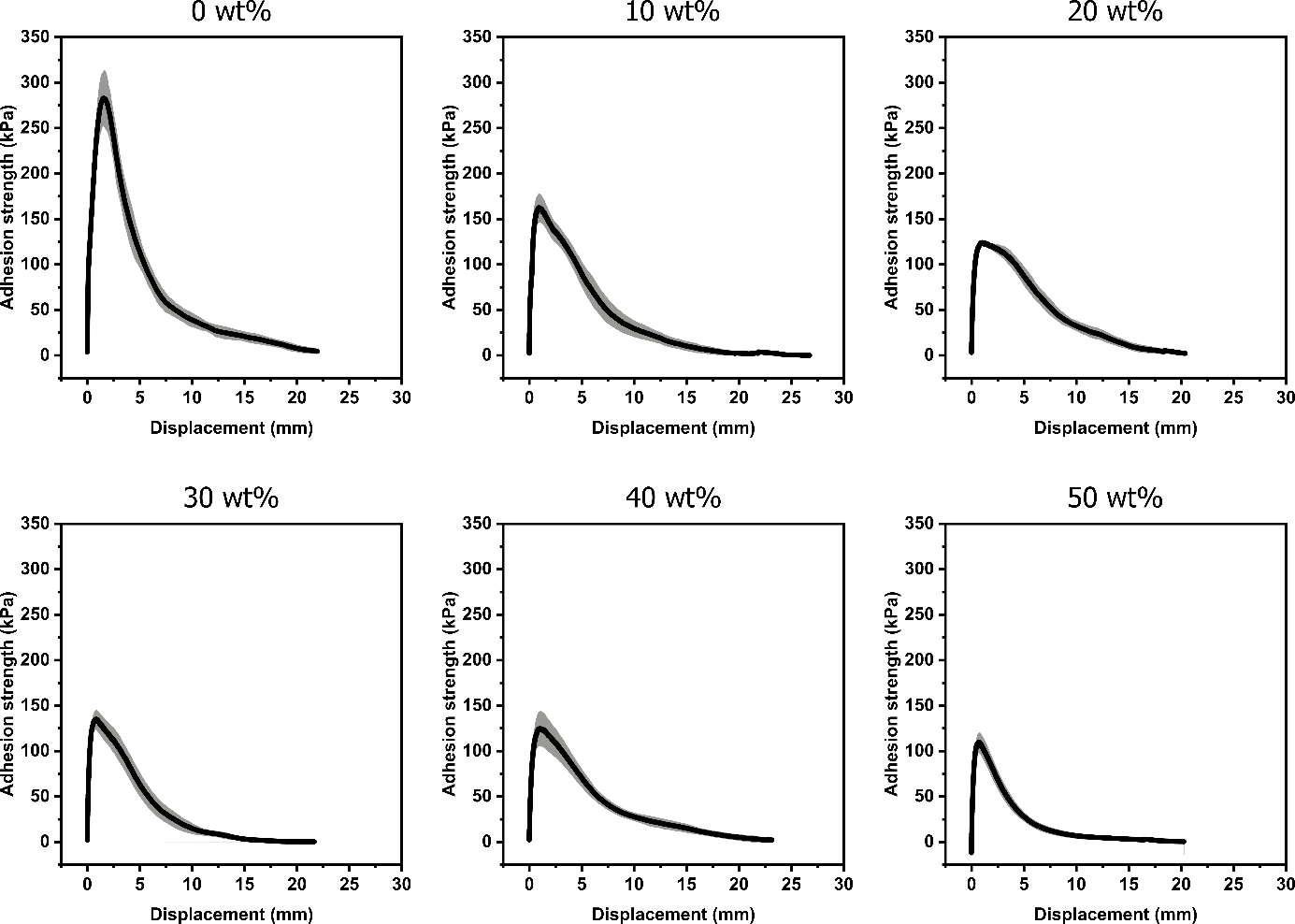


**Figure S9:** Comparison of the average lap-adhesion strength of neat PVAc film and PVAc/microcapsule composites (10-50 wt %) films (grey shaded: average ± standard deviation. The specimens were prepared with an adhesive thickness of ca. 600 µm and an area of 25 mm (width) x 15 mm (length).


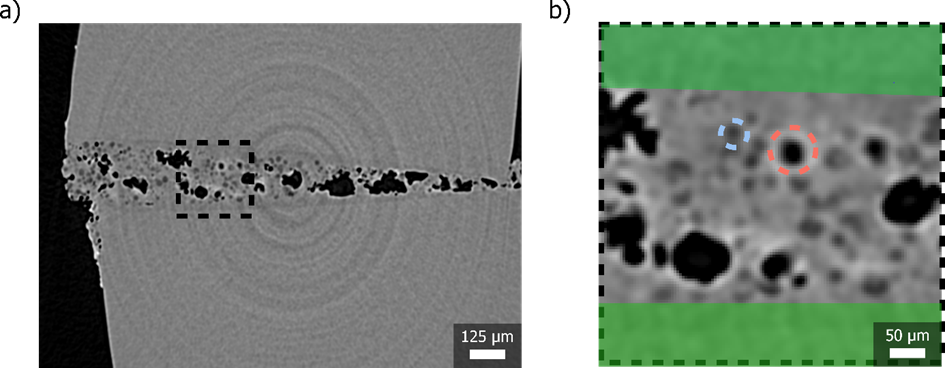


**Figure S10:** **a)** X-ray microtomography image of an adhesive composite containing 50 wt% capsules bonding two PMMA substrates. (a) Overview of the interfaces between the adhesive and the substrates. **b)** Magnified view of (a). Black areas represent air bubbles, grey areas indicate microcapsules filled with hexyl acetate, light grey areas correspond to PVAc, and PMMA substrates is highlighted in green.


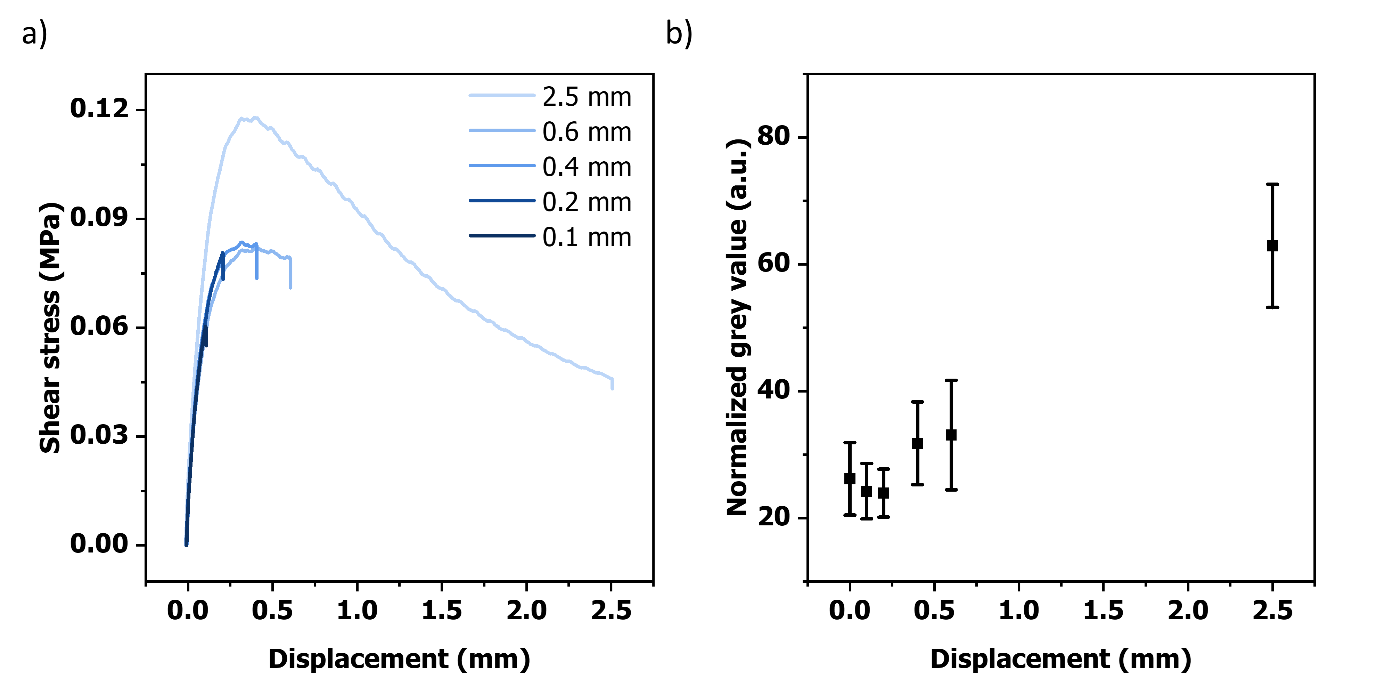


**Figure S11:** **a)** Displacement curves for composites containing 50 wt% MCs measured during lap shear tests. **b)**Plot of the normalized grey value obtained from RGB analysis of the specimens shown in (a). The RGB analysis was performed one week after the shear experiment described in (a).

*
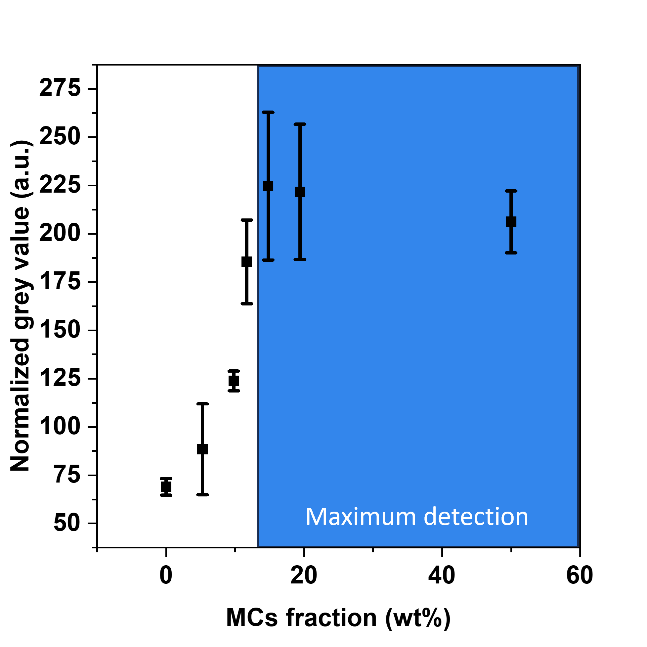
*

**Figure S12:** Plot of the normalized grey value obtained from RGB analysis of images captured for PVAc and composites loaded with 0 to 50 wt% MCs after compression under a 5 kN force.


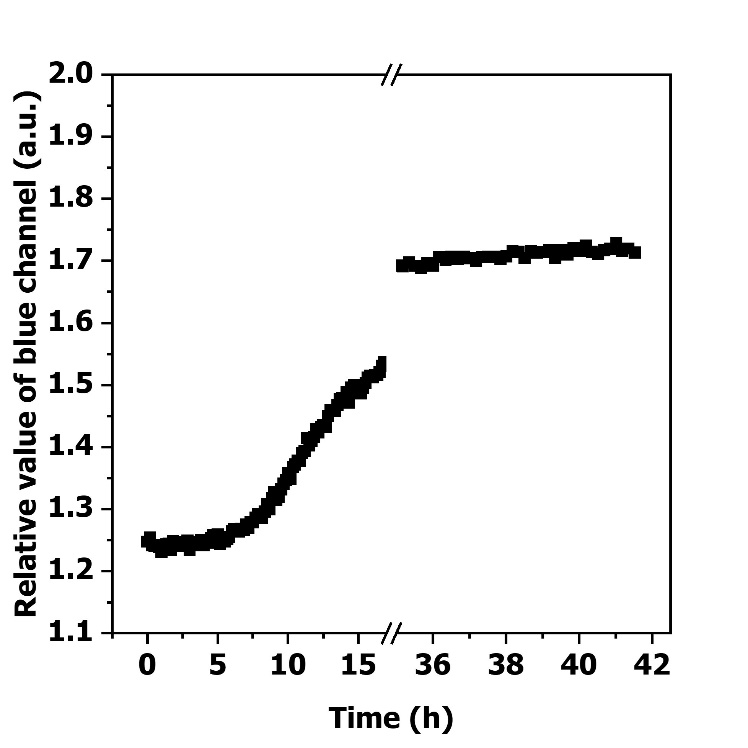


**Figure S13:** Change in the blue channel values as a function of time, derived from RGB analysis of images of the composite containing 50 wt% MCs. The images were captured after compression with a force of 5 kN applied for less than 10 seconds. Note that the extracted blue channel values were averaged in a given area and subsequently divided by the averaged grey value in the same area. See more details in the supporting method section.

**
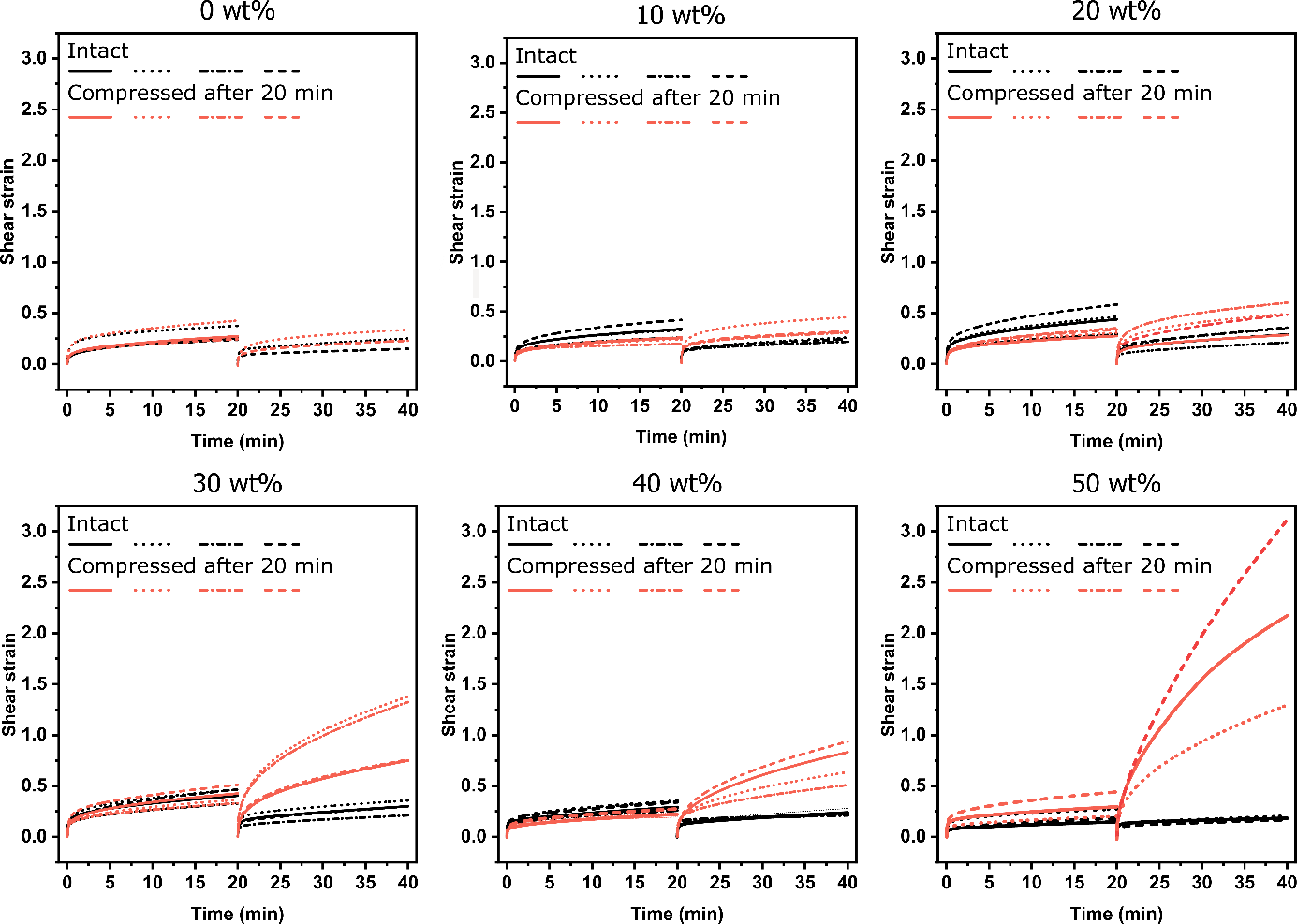
**

**Figure S14:** Comparison of individual creep experiments of neat PVAc film and PVAc/microcapsule composites (10-50 wt %). The creep experiment was performed for 20 min afterwards the measurement, part of the samples were compressed with a force of 5 kN (Compressed for less than 10 s). Afterwards a second creep experiment was performed. As a reference sample where not compressed before measurement (intact). Films were prepared with a thickness of ca. 600 µm and an area of 25 mm (width) x 15 mm (length).

**
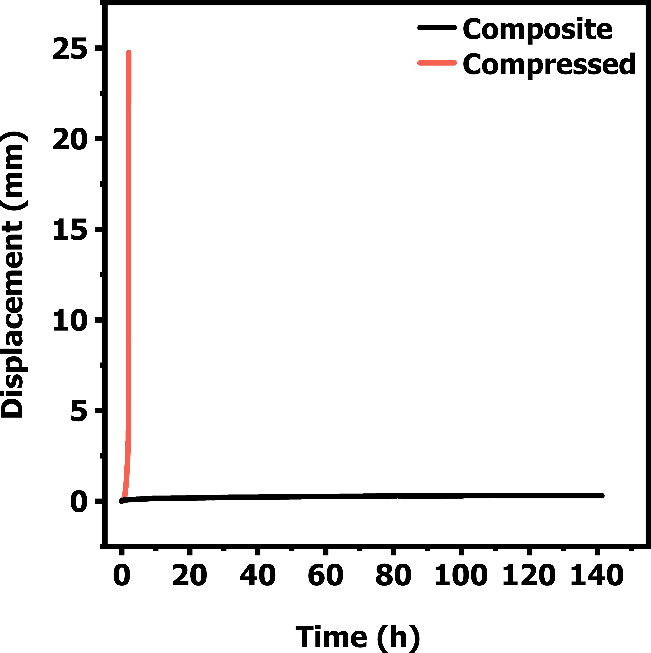
**

**Figure S15:** Plot of the displacement as a function of the time, obtained from a creep experiment of the neat PVAc and PVAc/MCs composite loaded with 50 wt% MCs. All creep experiments were conducted at room temperature with a mechanical load of 2.5 kN applied at a rate of 1 mm/sec.


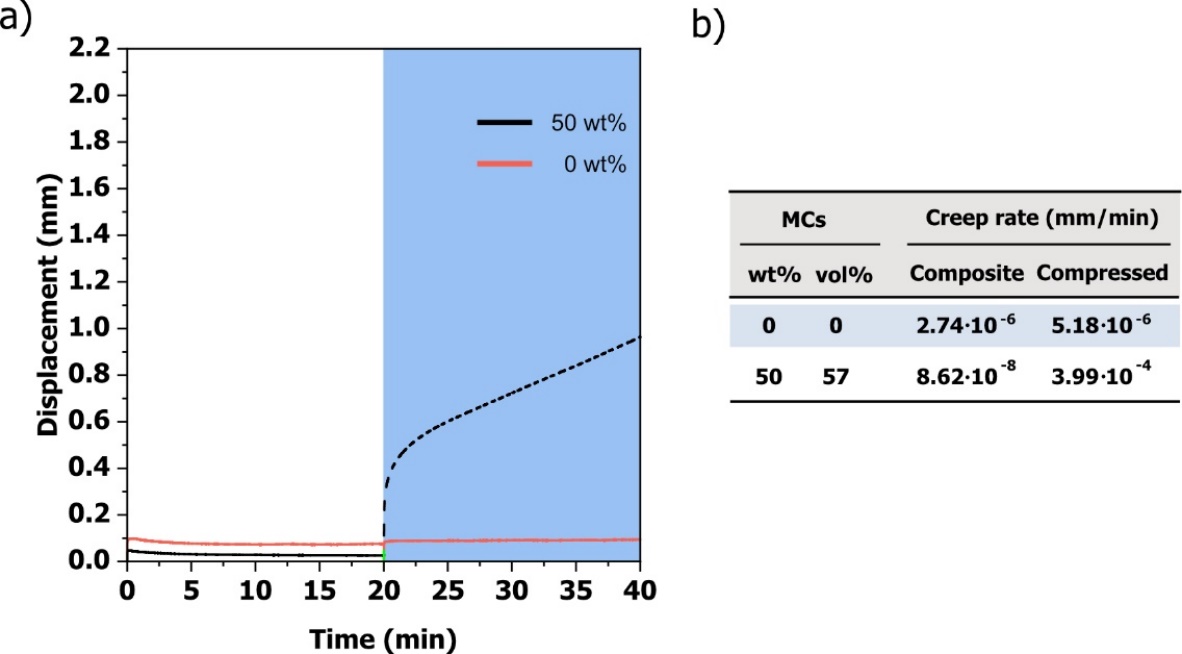


**Figure S16:** **a)** Plot of the displacement as a function of the time obtained from creep experiments on neat polychloroprene and polychloroprene/MCs composites embedded with 50 wt% MCs. The blue area depicts the creep behavior of the same samples after undergoing a 20-minute creep experiment and then being subjected to a compressive force of 5 kN for < 10 sec. **b)** Table of creep rates for neat polychloroprene and polychloroprene/MCs composites embedded with 50 wt% MCs, measured before and after compression with a 5 kN force

# Supporting Experimental Section

## Materials and Instrumentation

**Materials**

Ethylene-maleic anhydride (EMA) (M*_W_* = 100.000 – 500.000 g/mol), urea, resorcinol, ammonium chloride, hexyl acetate (99%), 1-octanol, ethanol and isopropanol were purchased in analytical grade quality from Sigma-Aldrich. Formaldehyde in solution (37% w/w in H_2_O) and n-hexane (≥ 99%) were purchased from Carl Roth. 1,1,2,2-Tetraphenylethylene (TPE) was purchased from TCI Chemicals. Ethyl acetate (≥ 99.8%) was purchased from Fisher Scientific. Desmodur L75(aromatic polyisocyanate based on toluene diisocyanate, equivalent weight: 315 mg, isocyanate-amount: 13.3 ± 0.4 %), was provided as a sample from Covestro. The PVAc based adhesive (UHU Alleskleber) was provided from Bolton Adhesives and the polychloroprene based adhesive (Pattex Kraftkleber Classic) was provided from Pattex Store. Poly methylmethacrylate (PMMA) sheets (thickness: 3 mm ± 0.200 mm) were purchased from Modulor. All chemicals and materials were used without further purification.

**Ultraviolet/Visible Light Absorption Spectroscopy**

UV-vis spectra were recorded on a Shimadzu UV-1800 spectrophotometer and data were acquired directly from the instrument in a CSV format. The substrates and films were measured in the absorption range from 200‑600 nm, with 2 nm steps at a slow scan rate.

**Fluorescence Spectra**

Fluorescence spectra of solid samples were recorded with an Ocean Optics Spectrometer 600 spectrometer (350-1040 nm, 1.65 nm Res.) under excitation at λ_ex_ = 365 nm from an Ocean Optics LS-450 LED light source using a 200 µm Reflection Probe (UV/VIS, 2 m) in reflection geometry. The samples were placed on a substrate and the optical fiber was oriented orthogonal to surface at a distance of 1 cm, resulting in a spot size of approximately 800 µm from which the diffuse reflectance was measured. Spectra were recorded before and after the application of mechanical stress and data were acquired with Stream basic software.

**Optical and Fluorescence Microscopy**

Micrographs were acquired using a Nikon SMZ 25 stereomicroscope and a Zeiss Observer Z1 microscope. The Nikon SMZ25 is equipped with a SHR Plan Apo 0.5x and 2.0x lens and a DS-Qi2 digital camera. Fluorescence microscopy images were acquired on the same microscope using a DAPI-filter (EX: 378/52; DM 409; BA 432). Photographs were taken with NIS-elements software and size measurements of the microcapsules (MCs) were carried out using the ImageJ software.^[1]^ The Zeiss Observer Z1 is equipped with EC Plan-Neofluar 5x/0.16 and 10x/0.30 lens and an Axiocam 506 color camera. Micrographs were acquired with the ZEN software.

**Photography**

Photographs were taken with an iPhone 13 or a Nikon ZFC digital camera equipped with a NIKKOR Z DX 18–140 mm lens under bright light or under a UV-light irradiation at 365 nm.

**Focused Ion Beam / Scanning Electron Microscopy (FIB/SEM)**

The instrument used is a Thermo Fisher Scientific DualBeam FIB/SEM Scios 2 HiVac with a Ga source for the ion beam and a FEG gun for the electron beam. For the process cross section, the sample was first tilted to the standard 52° angle. A platinum protective layer was deposited on the surface prior to ion beam cutting. The ion beam parameters used for cross sectioning were 30 kV and 7 nA for rough cross sectioning and 0.1 nA for fine milling.

**X-ray microtomography**

The X-ray microtomography measurements and data processing were performed using a BRUKER SkyScan 1272 and NRecon 3D reconstruction software. The x-ray power for the X-ray microtomography was set to 60 kV and the pixel size was set to 3 µm/pixel with an image size of 768X768 pixels.

**Preparation of** **Poly(urethane)/Poly(urea-formaldehyde) Microcapsules**

The preparation of poly(urethane)/poly(urea-formaldehyde) (PUF) MCs was performed following previously reported procedures.^[2,3]^ In a 100 mL two neck round-bottom flask, urea (0.83 g), resorcinol (83 mg), and ammonium chloride (83 mg) were mixed in 42 mL solution of 0.5 wt% EMA/H_2_O. Two drops of 1-octanol were added directly after mixing. In a separate Erlenmeyer flask, a 0.8 wt% TPE solution in hexyl acetate (158 mg in 20 mL) was prepared and stirred at 80°C for 15 min, until complete dissolution of the TPE. The solution was cooled to room temperature and mixed with Desmodur L75 (670 mg). The solution was then slowly poured into the round-bottom flask, containing the previously prepared aqueous solution, under mechanical stirring (Phoenix Instrument RSO 20A equipped with an overhead stirrer shaft featuring two movable paddles (diameter: 60 mm)) with a gradual rpm (revolutions per minute) increase from 450 to 850 rpm, and the flask was closed with a septum. After the formation of an emulsion following 10 minutes of mechanical stirring at 850 rpm, 2.1 g of formaldehyde was added to the mixture. The temperature was raised to 55 °C and the reaction mixture was stirred at 850 rpm for 4 h. After 4 h of stirring, the mixture was cooled to room temperature before being transferred to a 1000 mL separatory funnel. Subsequently, 300 mL of deionized water was added to the funnel. The contents were gently mixed by shaking the funnel to combine the product mixture with the water. The mixture was then left in the separatory funnel overnight, resulting in the formation of two distinct phases: an upper phase consisting of the MCs dispersion and a lower phase containing polymer fragments. The procedure was repeated two times with 700 mL of deionized water and a separation time of approximately 4 h. After washing the separatory funnel was refilled with approximately 400 mL of deionized water and sieved using a mesh of 680 μm × 365 μm.

An additional separation step was performed with the sieved dispersion before the MCs where filtrated and washed with water using a glass frit (porosity 4)

**Characterization of the Microcapsules**

Optical microscopy was used to measure the MCs’ average diameter. The average diameter and its standard deviation were determined by analyzing one image of a minimum 50 capsules using the software ImageJ.^[1]^ The weight fraction of the shell was determined by mechanically crushing the microcapsules (1 g) using a mortar and pestle. The crushed capsules were placed in a beaker with ethyl acetate (50 mL), the shell suspension was filtered, and thoroughly washed with ethyl acetate. After drying at ambient conditions for 1 day, the shell fragments were weighed (100 mg) and a filling content of 90 wt% was determined.

**Thermal Characterization.**

Differential Scanning Calorimetry (DSC) experiments were carried out under N_2_ atmosphere on a Netzsch 204 F1 Phoenix DSC instrument. All samples were annealed at 70 °C for at least 3 h prior to measuring and after cutting the films. Samples having a weight of around 5 to 15 mg were heated from -90 to 90 °C with heating and cooling rates of 10 °C min^−1^. DSC samples of compressed neat PVAc, and PVAc/MCs composite films were measured after compressing by a force of 5 kN (< 10 s). Thermogravimetric Analysis (TGA) experiments were carried out under N_2_ atmosphere on a NETZSCH STA 449 F5 thermo-gravimetric analyzer. Samples were heated from 25 to 180 °C with a rate of 5 °C min^−1^, held at 180 °C for 2 hours and heated from 180 to 600 °C with a rate of 5 °C  min^−1^.

**Preparation of Neat PVAc and PVAc/Microcapsule Composite Films**

The PVAc glue was thinned with approximately the same amount of ethyl acetate. To form PVAc/MCs films, the PVAc/ethyl acetate solution was blended with MCs at different weight fraction ranging from 10 to 50 wt% (relative to the dry mass of PVAc). Subsequently, the neat PVAc and PVAc/MCs solutions were solvent-cast into PTFE dishes and dried under ambient conditions for 3 days. Afterward, the samples were dried under vacuum overnight.

**Preparation of Single Lap Joint Specimen**

Single lap joints were prepared for colorimetric readout characterization using microscopic glass slides (100 mm × 25 mm ×1 mm) and for mechanical measurements using PMMA sheets (*d* = 3 ± 0.200 mm). The microscopic glass slides were used as received. The PMMA sheets were lasercutted (maitech lasercutter: 1 pass, 45 % intensity, 15 mm/s) to dimensions of 25 mm × 100 mm, before the safety foil of the PMMA was removed. The adhesive was mixed with MCs in a small vial using a spatula to form the PVAc/MCs composites. Note that the required amount of MCs to achieve the desired wt%-loading was calculated based on the adhesive's solid content of 36 wt%. The prepared composite was applied with a syringe (*V* = 0.3 – 0.5 mL) on the marked bonding area (length = 15 mm, A = 375 mm^2^) of one slide (Figure S12 1). It was spread with a spatula and let become touch dry for 5 to 10 minutes (Figure S12 2-3). The second slide was placed on the bonding area of the first slide and supported by a spacer according to the slide thickness (Figure S12 4). Pressure was applied by hand on the upper slide and the slides were aligned to each other in a parallel manner. The specimen was dried at ambient conditions for 6 days.

Note: The same procedure was applied for the preparation of lap joint specimens bonded with Pattex glue (polychloroprene matrix) with an adhesive’s solid content of 23 wt%.


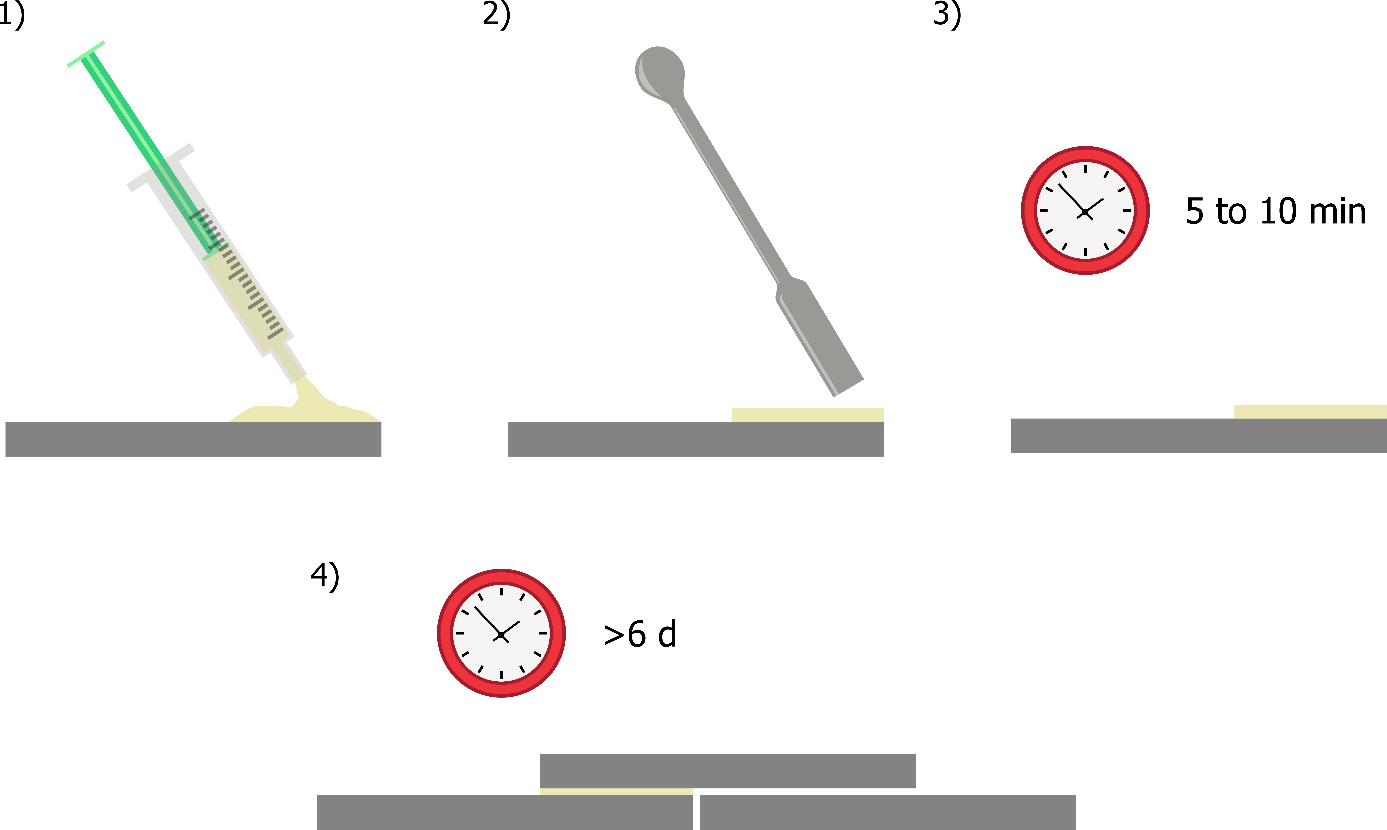


**Figure S17:** Preparation of single lap joint specimens. Single lap joints were formed by **1)** applying the PVAc/microcapsule to a substrate using a syringe, **2)** and distributing the adhesive evenly with a spatula. **3)** After 5 to 10 min the PVAc/microcapsule composite was touch dry **4)** and the second substrate was applied on top of the PVAc composite.

**Colorimetric Readout Characterization (RGB-analysis).**

Specimens were photographed under UV irradiation (λ_exc_ = 365 nm) to capture their fluorescence response. Image acquisition was performed using a standard digital camera setup (described in the section: Photography). For quantitative analysis, the resulting images were processed in the open-source software ImageJ.^[1]^ Specifically, five distinct regions of interest (ROI) were selected on each specimen, arranged in a quincunx pattern to ensure representative sampling across the surface. Within each ROI, the mean pixel intensities for the red, green, and blue (RGB) channels were extracted. From these measurements, either (i) the average intensity of the blue channel alone or (ii) the mean gray value (averaged across all three channels) was calculated for each specimen. Finally, to account for variability in adhesive layer thickness, exposure time, and light source intensity, all measured intensities were normalized by dividing by the adhesive thickness and multiplying by a defined scaling factor.

**Mechanical Testing.**

Compression measurements were performed at ambient conditions using a uniaxial testing machine (Zwick/Roell testControl II) equipped with a 10 kN load cell. The bonding areas of the single lap joint specimen were photographed before and after mechanical measurements to monitor their occurrence. The thickness of the bonding areas was measured before and after compression. A maximum compressive force of 5 kN was applied up to the bonding area of one specimen with a compression rate of 10 % min^-1^ with subsequent unloading to 0 N. A typical compression process takes < 10 s.

Lap shear strength and creep measurements were performed at ambient conditions using a tensile tester (Hegewald & Peschke inspect table 5) equipped with a 1 kN load cell. The initial grip-to-grip separation was 112.5 mm and the grips were installed with a small offset to each of 3 mm according to the single lap joint geometry.

The lap shear strength measurement was performed with a speed of 5 mm/min (Figure S13a) and manually stopped when the recorded force was < 0.4 N. The occurrence of the bonding areas was documented by photographs. The shear strain is defined as the recorded displacement divided by the thickness of the adhesive respectively the MC/adhesive composite joining the substrates. The stress was calculated by dividing the recorded force with the bonding area of 375 mm^2^. The lap adhesion strength was extracted as the maximum of the stress (Figure S13 b), the shear modulus was determined by a linear regression of the initial shear stress-strain curve (Figure S13 c, 0 to 0.01 mm displacement), the toughness was determined by integrating the area under the stress-strain curve (Figure S13d), the energy until failure was determined by integrating the area under the stress-strain curve until the shear stress reaches the lap shear strength (Figure S13e).


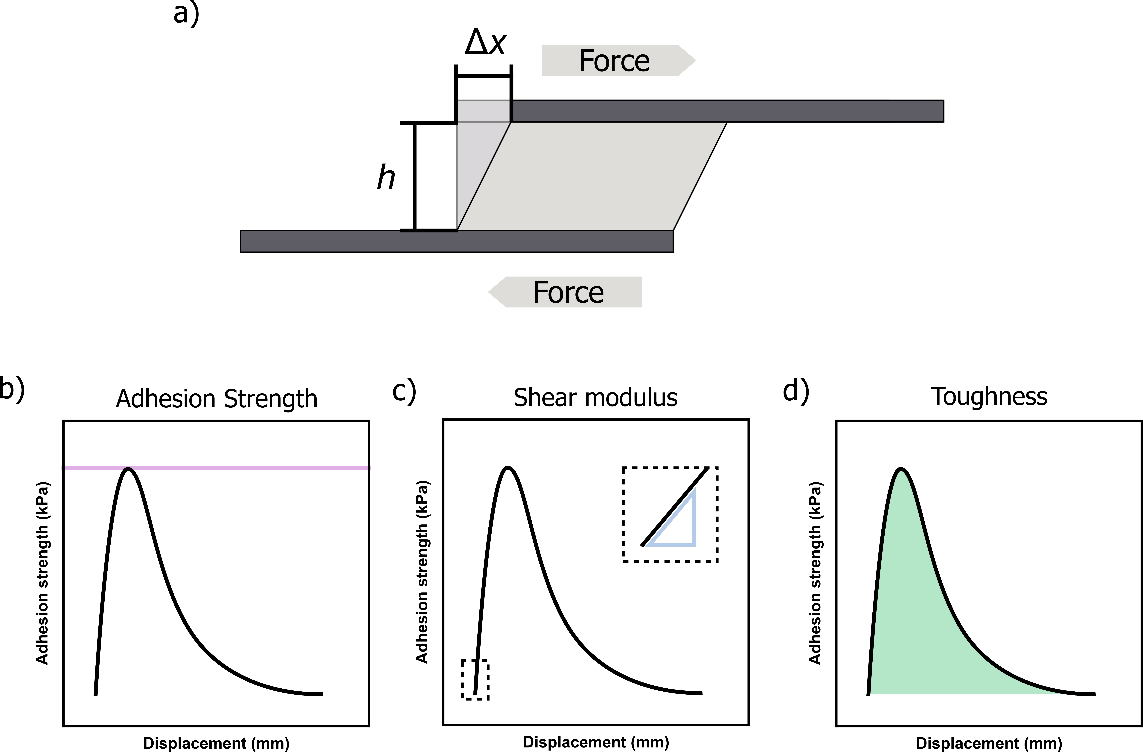


**Figure S18: a)** Schematic depiction of a single lap joint which is subject to a shear force causing a shear strain of γ=Δx/h. **b-e)** Schematic depiction of stress-strain curve which depict **b)** Adhesion strength, **c)** shear modulus, **d)** toughness.

To perform a typical creep measurement the sample was elongated with a speed of 1 mm/min until a load of 2.5 N was reached from that point, the force was held for 20 min (Figure S14 a). The specimen was clamped out of the grips, either compressed (5 kN, with 10% min^-1^, typically <10 s) or not and again inserted. The creep measurement was then performed for another 20 min under the same parameters. The creep rate was determined by a linear regression of the second stage of the creep experiment (Figure S14b). The occurrence of the bonding areas was documented by photographs. The shear strain was calculated by dividing the recorded displacement with the thickness of the adhesive respectively the MC/adhesive-composite joining the substrates.


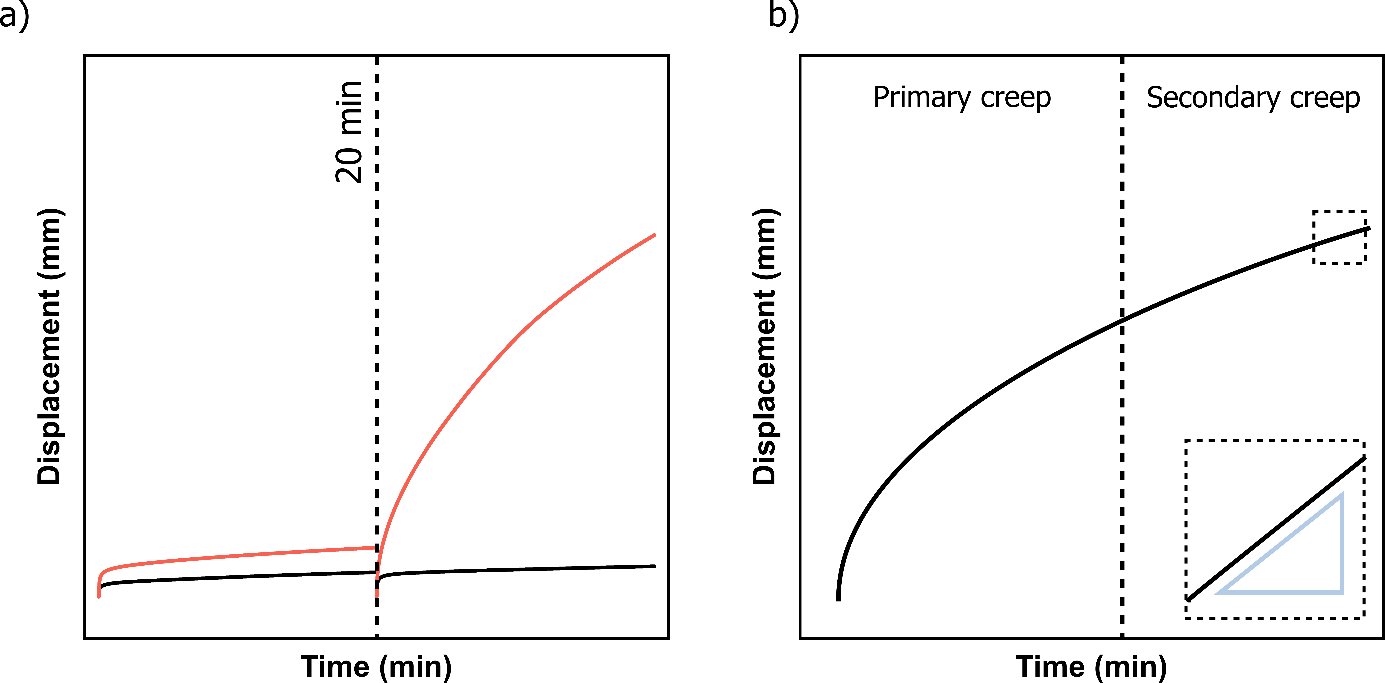


**Figure S19: a)** Schematic depiction of a creep experiment. **b)** Schematic depiction of the determination of the creep rate.

# References

[1] C. A. Schneider, W. S. Rasband, K. W. Eliceiri, *Nat Methods* **2012**, *9*, 671.

[2] M. J. Robb, W. Li, R. C. R. Gergely, C. C. Matthews, S. R. White, N. R. Sottos, J. S. Moore, *ACS Cent Sci* **2016**, *2*, 598.

[3] M. M. Caruso, B. J. Blaiszik, H. Jin, S. R. Schelkopf, D. S. Stradley, N. R. Sottos, S. R. White, J. S. Moore, *ACS Appl Mater Interfaces* **2010**, *2*, 1195.
